# Efficacy of fermented foods for the prevention and treatment of bacterial vaginosis and vulvovaginal candidiasis

**DOI:** 10.3389/fnut.2025.1658988

**Published:** 2025-11-21

**Authors:** Aslı Akpınar, Lidia Hanna Markiewicz, Hayriye Şebnem Harsa, Diana Paveljšek, Julieta Domínguez-Soberanes, Zeynep Agirbasli, Eleni Naziri, Mounaim Halim El Jalil, Gregory Bouchaud, Seppo Salminen, Isabelle Savary-Auzeloux, Christèle Humblot, Christophe Chassard, Smilja Pracer, Guy Vergères, Barçın Karakaş-Budak

**Affiliations:** 1 Department of Food Engineering, Faculty of Engineering and Natural Science, Manisa Celal Bayar University, Manisa, Türkiye; 2 Immunology and Food Microbiology Group, Institute of Animal Reproduction and Food Research, Polish Academy of Sciences, Olsztyn, Poland; 3 Department of Food Engineering, Izmir Institute of Technology, Izmir, Türkiye; 4 University of Ljubljana, Biotechnical Faculty, Ljubljana, Slovenia; 5 Facultad de Ingeniería, Universidad Panamericana, Aguascalientes, Mexico; 6 Department of Food Science and Nutrition, School of Environment, University of the Aegean, Mytilene, Greece; 7 Ecole Supérieure de Technologie, Mohammed V University of Rabat, Rabat, Morocco; 8 INRAE, Biopolymères Intéractions Assemblages (BIA), Nantes, France; 9 Center for Nutrition and Food Research, Faculty of Medicine, University of Turku, Turku, Finland; 10 Université Clermont Auvergne, INRAE, UMR1019 Nutrition Humaine, Saint Genès Champanelle, France; 11 QualiSud, Université de Montpellier, Avignon Université, CIRAD, Institut Agro, IRD, Université de la Réunion, Montpellier, France; 12 French National Research Institute for Sustainable Development (IRD), Montpellier, France; 13 INRAE, Unité Mixte Recherche sur les Fromages, VetAgroSup, Aurillac, France; 14 Institute for Biological Research Sinisa Stankovic, National Institute of Republic of Serbia, University of Belgrade, Belgrade, Serbia; 15 Research Division Microbial Food Systems, Agroscope, Berne, Switzerland; 16 Department of Food Engineering, Akdeniz University Faculty of Engineering, Antalya, Türkiye

**Keywords:** vaginosis, vaginal microbiome, fermented dairy food, probiotic starter, fermented foods, woman, female genital system, systematic review

## Abstract

Vaginal function in healthy women is closely associated with a lactobacilli-dominated microbiome. Among the most common conditions arising from dysbiosis are bacterial vaginosis (BV) and vulvovaginal candidiasis (VVC). While the efficacy of oral probiotics for the treatment of BV and VVC is well documented, the role of consuming fermented foods remains underexplored. This systematic review aims to present a systematic evaluation of the potential role of fermented foods in the prevention and treatment of BV and VVC and establish the extant research gap between the realm of the clinical sciences and the field of food science and technology. For this purpose, under the guidance of COST Action CA20128—Promoting Innovation of Fermented Foods (PIMENTO), a systematic literature review was conducted in two phases. PubMed, Scopus, and Cochrane databases were used for Phase I to analyze articles on human trials and observational studies where the intervention/exposure involved oral consumption of fermented food. In Phase II, a two-step search strategy was employed: (i) identifying microorganisms with demonstrated clinical efficacy in managing BV and VVC, and (ii) reviewing food science literature where these strains are utilized for fermentation. It was observed that 87% of the food starter applications exploited only two of the 54 efficacious strains identified through clinical studies, namely *Lactobacillus rhamnosus* GG and *Lactobacillus acidophilus* LA-5. Findings underscore the potential of fermented foods as carriers for beneficial microorganisms and their relevance in supporting vaginal health. This review contributes to a deeper understanding of the interplay between nutritional consumption of viable probiotic strains and their importance in immunomodulation, highlighting the need for more integrated research efforts across disciplines. Future research aimed at filling this gap will enable informed clinical decisions and dietary recommendations.

## 1 Introduction

The human vaginal microbiome is a crucial site of symbiosis where lactobacilli rule the microbial community and protect women from infectious diseases across their lifespan (1). Changes in the vaginal microbial population can result in dysbiosis where the fast decline in microbial diversity encourages the growth of detrimental non-*Lactobacillus* species. Some of these bacteria or yeast strains may trigger immune responses and ultimately increase susceptibility to infections and contribute to negative reproductive outcomes (2). Vaginal dysbiosis is the result of imbalances in the vaginal microbiota often the root cause of vaginitis characterized by an abnormal vaginal milieu and leading to vaginal symptoms and signs. Bacterial vaginosis (BV) and vulvovaginal candidiasis (VVC) are the two most prevalent types of vaginitis. In some cases, mixed infections with simultaneous characteristic expression of both BV and VVC may occur (3).

Bacterial vaginosis (BV) is a polymicrobial disorder characterized by a shift in the composition of the vaginal microbiota, where there is a decrease in beneficial *Lactobacillus* species and an overgrowth of infectious bacteria, particularly anaerobic bacteria such as *Gardnerella vaginalis*, *Prevotella* spp., and others (4, 5). Bacterial vaginosis is associated with multiple adverse gynecologic and obstetric outcomes, including pelvic inflammatory disease (PID) and an increased risk of preterm birth in pregnant women. While BV is not considered a sexually transmitted infection (STI), it can increase the risk of acquiring certain STIs, such as human immunodeficiency virus (HIV), herpes simplex virus, and chlamydia. The treatment regimen may vary depending on the severity of the infection, individual patient factors, and the healthcare provider’s preferences. Usually, antibiotics are prescribed to eliminate the overgrowth of infectious bacteria. In addition to antibiotic treatment, some healthcare providers may recommend the use of vaginal probiotics or oral probiotics to help restore and maintain a healthy vaginal microbiota.

*Candida albicans* is a fungus within the human mycobiome identified in the vagina of a significant portion of asymptomatic (healthy) women (6). The opportunistic nature of the yeast *Candida albicans* (and other *Candida* sp.) may result in an overgrowth and cause a state of dysbiosis referred to as vulvovaginal candidiasis (VVC). Antibiotic use, glucocorticoid use, hormonal changes, uncontrolled diabetes, pregnancy, or immunosuppression are risk factors for VVC. The care provider usually prescribes antifungal medication (oral or intravaginal) targeting the overgrowth of *Candida*. Management of risk factors are also important for the inhibition of recurrent VVC (RVVC).

It is important that the microbiota of the female genital tract is kept in balance to ensure immune function (7). Increasing number of studies establish that administration of oral probiotics in the form of supplements is effective in the prevention and treatment of BV and VVC (8, 9). These studies make use of probiotic strains of *Lactobacillus* and/or *Bifidobacterium* which when taken orally help restore endogenous vaginal microflora by competitively, biochemically, and immunologically replacing pathogens. Yet, while probiotic supplements have been extensively studied, fermented foods merit exploration as they may offer a more sustainable and culturally integrated means of delivering beneficial microorganisms that support vaginal health.

The PIMENTO initiative is a COST Action focused on the health benefits and risks of fermented foods (10). One of the aims of PIMENTO is to establish the grounds for claims related to the efficacy of fermented foods for maintaining immune function and emphasizes the importance of preserving this functionality via the consumption of fermented foods. Consumption of fermented foods containing efficacious *Lactobacillus* and *Bifidobacterium* spp. may potentially be beneficial for immune function of the vagina. This systematic review aims to address this gap and focus on the potential role of fermented food in the diet to modulate vaginitis by answering the question: “Can consumption of fermented foods prevent bacterial vaginosis or vulvovaginal candidiasis?” in order to provide a comprehensive assessment on the available evidence for the efficacy of fermented foods for prevention of or recovery from BV and/or VVC. This systematic evaluation is accompanied by a narrative description of product characteristics, mechanism of action and safety. Additionally, an innovative approach of the review was to screen efficacious probiotic strains from literature (i.e., to compile a list of specific microbial strains that when administered orally have been shown to be efficacious against stated clinical indications) which was then used to perform a systematic search in order to establish the use of these probiotic microorganisms for the production of fermented foods. For this purpose, the review has been structured in two phases; Phase I (identification and evaluation of human studies for investigating efficacy of fermented foods against BV and VVC) and Phase II (cataloging efficacious probiotic strains used in oral intervention against BV/VVC and identifying food science and technology studies utilizing these for food fermentation).

## 2 Methods

This review was performed in accordance with recently published guidelines (11). The protocol was registered in Open Science Framework (OSF) (12). The searches were performed in two sections (Phase I, Phase IIa and Phase IIb). For the first section (Phase I), systematic searches were limited to articles published within 1.1.1970–31.12.2024 (initial search performed until 31.08.2023 and updated until 31.12.2024 per PIMENTO protocol) and for the second section (Phase II) the searches were limited to articles published within 1.1.1970–11.04.2025. Only articles in English were included and assessment of eligibility was achieved in duplicate using the CADIMA tool (13) by two reviewers assigned randomly. Discrepancies were resolved through discussion until consensus was reached.

### 2.1 Phase I: Human studies for investigating efficacy of fermented foods against BV and VVC

The generic search strategy developed within the PIMENTO initiative was employed (Phase I). This strategy and strings used have been published in a position paper (10). Briefly, the generic search aimed to compile literature (interventional and observational studies) where the intervention (I) against BV and/or VVC involved ingestion of fermented food. Therefore, strings related to the conditions of BV and VVC were adopted for the present review (Supplementary section S1.1). The population (P) was female subjects (women) of/after reproductive age (13 + years of age), including menopausal women, pregnant women, nursing women. PubMed, Scopus and Cochrane Library database search results were evaluated for P/I and outcome (O) criteria to extract eligible publications (Supplementary section S1.2). Comparator (C) was defined as any intervention that did not contain viable fermentation strains and only evaluated at data extraction level. Only original research articles were used for data extraction and reviews were retained to check for eligible articles within the list of references.

The studies selected on the basis of the P/I/O criteria, i.e., studies that included a relevant population, investigated a specific intervention and reported clinically meaningful outcomes, were assessed for methodological quality and risk of bias according to their design using standardized tools. Randomized controlled trials (RCTs) were evaluated using the revised Cochrane Risk of Bias Tool (RoB2.0; randomized studies) (14), which assesses bias related to the randomization process, period and carryover effects (in cross-over trials), deviations from intended interventions, missing outcome data, measurement of outcomes and selection of reported results. Each domain was rated individually and an overall judgment on risk of bias was made for each study. Non-randomized studies (observational studies) were assessed using the modified Newcastle-Ottawa Scale (NOS; non-randomized studies) (15, 16). This tool evaluates studies in three domains: selection of the study group, comparability between responders and non-responders, and either exposure (for case–control studies) or outcome assessment (for cross-sectional studies). Each study could receive a maximum of 7 or 9 stars, depending on its design. A rating of ≥4 stars (7-point scale) or ≥5 stars (9-point scale) was considered the threshold for good methodological quality. Risk of bias assessments were performed independently by two reviewers. Discrepancies were resolved through discussion until consensus was reached.

Sections related to discussions of product characteristics, mechanism of action and safety have been supported with non-systematic narrative synthesis.

### 2.2 Phase II: Cataloging efficacious probiotic strains used in oral intervention against BV/VVC and identifying food science and technology studies utilizing these for food fermentation

In this section, two separate sequential searches were performed (Phase IIa and Phase IIb). The aim of the first search (Phase IIa) was to compile a list of microorganisms which were shown to be effective in reducing the symptoms of or curing from BV and/or VVC. To this end, specific strings of the PIMENTO search strategy were implemented to compile a list of strains from published research on human subjects (clinical trials) where the intervention was in the form of a probiotic supplement ingested orally. The string components related to the condition (i.e., BB/VVC) and databases included were the same as indicated in Phase I. Search strings and selection criteria are detailed in Supplementary sections S2.1, S2.2. Search results were evaluated for P/I O criteria to select articles and compile a list of efficacious microbial strains at the species and subspecies level (Supplementary section S2.3). In the subsequent search (Phase IIb), the compiled strain nomenclature was used to construct a search query to locate publications in the realm of food science and technology where the study involved production of fermented foods utilizing these specific strains as fermentation organisms. Articles were searched using a string (Supplementary section S3.1) designed to include all 54 strains (listed in Supplementary section S2.3). Articles were filtered using indexing tools to include only research articles published in the Food Science and Technology category of the Web of Science database (and selected as detailed in Supplementary section S3.2).

## 3 Results and discussion

### 3.1 Study selection

**Figure 1:**
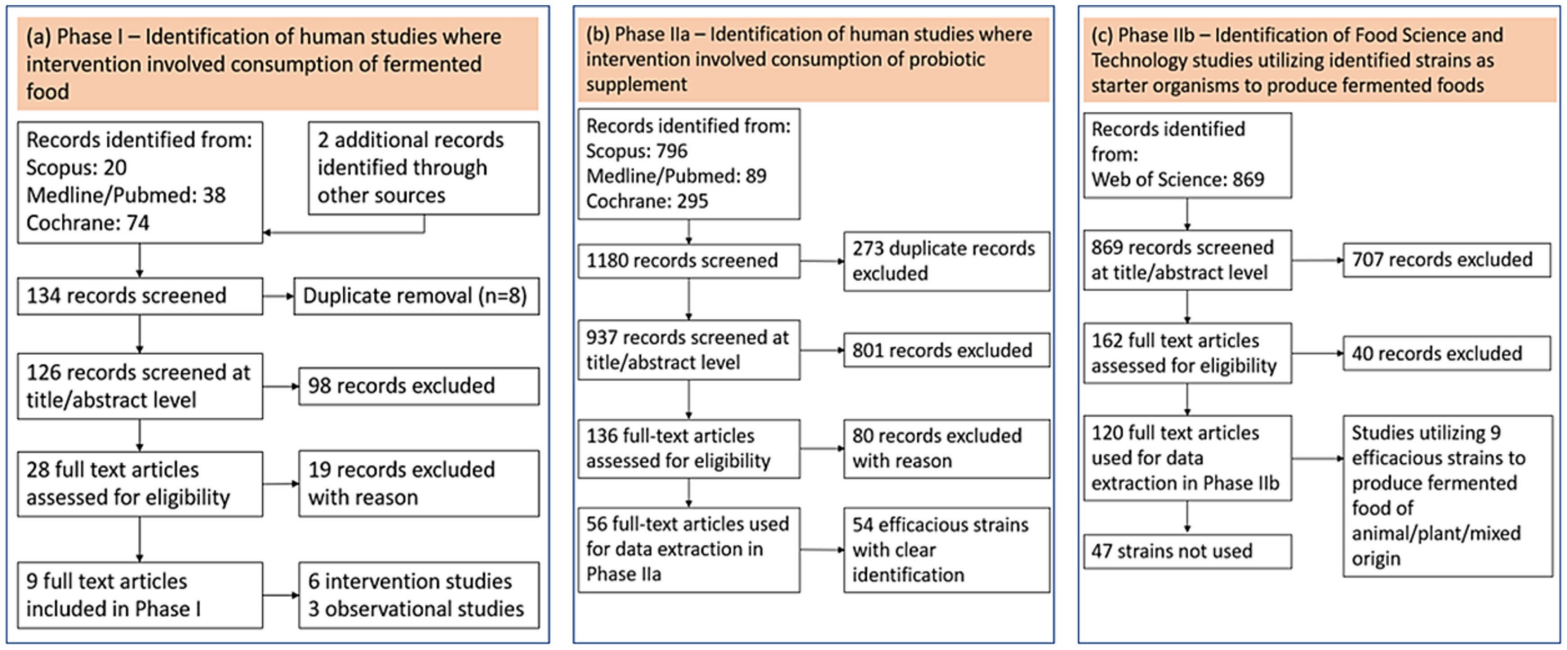
Study selection. **(a)** Phase I—identification of human studies on efficacy of fermented food against BV/VVC, **(b)** Phase IIa—identification of RCT studies on efficacious probiotic supplements, and **(c)** Phase IIb—screening of food science and technology research articles utilizing efficacious strains as fermentation starters.

Flow schemes explaining the selection of studies for different phases of the present review are presented in Figure 1. The aim of Phase I was to assess the state of the art related to efficacy of fermented foods against BV and VVC based on RCT’s and human observational studies. This assessment also detailed the characteristics of fermented foods, the mechanism of action and safety. The aim of Phase II was to identify efficacious probiotic strains consumed orally in supplement form (Phase IIa) and summarize research where they have been utilized as food fermentation organisms (Phase IIb).

### 3.2 Efficacy of fermented foods against BV and VVC—RCT’s and observational studies

#### 3.2.1 Results of interventional studies

The main findings of the clinical trials evaluating intervention with fermented foods against BV and/or VVC are summarized in Table 1. A total of 6 clinical studies (17–22) were identified, two were published in the 90’s and the remaining were published between 2011 and 2017. Articles contained variations in clinical aspects (P/I/O), methodology and evaluation. Due to the high heterogeneity of the data, conducting a meta-analysis was deemed infeasible. The concept of a gut-vagina axis is relatively new and contributes to new insight in our understanding of the function and immunology of the female genital system (23). However, it is interesting to note that trials involving intervention with fermented foods in recent years (more than 5 y) have not been published. Of the 6 RCT trials (3 articles BV, 2 articles VVC and 1 both), all had positive outcomes for prevention or treatment of BV and/or VVC as observed in the primary and secondary outcomes listed in Table 1. All articles presented statements that supported effects were positive. However, the studies also had limitations to some extent. Some of the articles were performed on a limited number of participants (19, 20), applied intervention for a brief duration (17), published as pilot RCT’s (22), short communication (21) or brief report (19). The first three publications (19–21) did not report the specific starter cultures employed in the fermentation process, which likely included *S. thermophilus* and *L. delbrueckii* subsp. *bulgaricus*, as these are conventionally utilized in yogurt production. Additionally, the first two studies (19, 20) did not provide strain-level identification for *L. acidophilus* cultures. Shalev (19) investigating effects for both BV and VVC reported only beneficial effects of yogurt consumption against BV. On the other hand, Dols et al. (21) observed beneficial effects against BV for both the intervention (yogurt with *L. rhamnosus* GR-1) and control group (consuming standard yogurt). Hantoushzadeh et al. (17), found no significant differences between treatments when comparing the efficacies of medical treatment (clindamycin) versus yogurt consumption against BV, indicating ingestion of yogurt containing probiotic bacteria could be as effective as conventional medical intervention. To complement efficacy evaluations, several studies have employed established diagnostic tools to objectively assess changes in vaginal microbiota and clinical symptoms. The Nugent score is a Gram stain scoring system for vaginal swabs to diagnose BV and the Amsel criteria provide an alternative assessment for BV diagnosis based the presence of at least three of four findings: vaginal discharge, elevated vaginal pH, clue cells on microscopy, and a positive whiff test. Both Dols et al. (21) and the most recent study by Laue et al. (18) reported no significant change in the Nugent scores. However, Laue et al. (18) also confirmed that symptomatic relief was significant as indicated by the differences in Amsel criteria scores for intervention and control groups.

**Table 1:** Clinical trials investigating the effect of ingestion of fermented food on BV and/or VVC^a^.

References	Study design	Disorder (BV/VVC)	Population/participants completing protocol	Treatment		Summary of outcome		Statement in publication
				Intervention (ingestion)	Control	Primary outcome	Secondary outcomes	
Hilton et al. (20)	Cross over trial	VVC	33 women entered the study, 13 women 24–50 years old, (mean age 35 years) completed the protocol	Yogurt containing *L. acidophilus* (10^8^ CFU^a^/ml), 8 oz./day for 6 months	No yogurt consumption for 6 months	VVC infections decreased by a factor of 3 in the intervention group (*p* = 0.001)	Candidal colonization decreased significantly (*p* = 0.001)	“Daily ingestion of 8 ounces of yogurt containing *L. acidophilus* decreased both candidal colonization and infection”
Shalev (19)	Cross over trial (brief report)	BV and VVC	46 women entered the study (20 BV, 18 VVC and 8 with both); 23 women were assigned to each arm of the trial (mean ages of intervention and control were 29 ± 6 and 31 ± 8, respectively); 7 women completed the protocol	Yogurt containing more than 10^8^ CFU/mL live *L. acidophilus*, 150 mL/day for 2 months	Pasteurized yogurt, 150 mL/day for 2 months	Significant reduction in BV episodes in intervention group after 1 and 2 months (*p* < 0.001); 6/7 patients that completed the study had 1–2 episodes of BV when on the control arm, compared to 1 episode of BV during intervention	Steady reduction in Candida(+) vaginal cultures in both groups, from 60% in the first month to 20–28% after 2 months; No significant difference in VVC between the 2 study periods in the 7 women who completed the study (*p* = 0.67)	“Daily ingestion of 150 mL of yogurt, enriched with live *L. acidophilus*, was associated with an increased prevalence of colonization of the rectum and vagina by the bacteria, and this ingestion of yogurt may have reduced episodes of BV”
Dols et al. (21)	Randomized, double blind study (short communication)	BV	145 HIV(+)^b^ women 25–73 years old, (mean age 40.5 years)	Yogurt containing *L. rhamnosus* GR-1, 125 mL/day for 29 days; some subjects were given HIV medication/antibiotics	Standard yogurt, 125 mL/day for 29 days	Rate of cure was 92% in the probiotic group and 89% in the standard yogurt group Nugent scores remained unchanged for 45–50% of all participants and improved for 38–39% of all participants (*p* > 0.1)	Subjects reported fewer vaginal symptoms and signs at day 29 compared to baseline; no differences found between those taking probiotic and regular yogurt; no significant changes in vaginal pH	“Yogurt provides a safe nutritious food that can be made locally and taken daily by HIV-subjects receiving anti-retroviral therapy. In total, 92% women in the probiotic group and 89% in the standard yogurt group did not have BV at the end of the trial”
Hantoushzadeh et al. (17)	RCT (double-blind, placebo-controlled, parallel-group)	BV	300 pregnant women divided into 2 groups to receive intervention or control; mean age and mean gestational age of each group was 29 years and 36 weeks, respectively	Yogurt containing *L. bulgaris*, *S. thermophilus*, probiotic lactobacillus, and *Bifidobacterium lactis*, 2 × 100 g/day for 1 week	Clindamycin, 2 × 300 mg/day for 1 week	BV cure rates were the same in both groups; 70–88% of all patients had decreased pH following treatment (*p* < 0.0001)	Preterm birth rates in the probiotic and clindamycin group were 8% (*n* = 12) and 5% (*n* = 7), respectively (*p* > 0.05). PROM^c^ rates were 6% (*n* = 9) in the probiotic group and 3% (*n* = 5) in the clindamycin group (*p* > 0.05). Symptom recurrence rates were 7% (*n* = 10) in the probiotic group 6% (*n* = 9) in the clindamycin group (*p* > 0.05)	“This study showed that probiotic yoghurt is as effective as the standard therapeutic regimen of clindamycin in the treatment of symptoms and prevention from recurrence in women with bacterial vaginosis in pregnancy”
Hu et al. (22)	Pilot study (non-randomized controlled trial)	VVC	24 women; 17 HIV(+) and 7 HIV(−);	Yogurt (DanActive™, containing *Lactobacillus casei*, 88 g/day or YoPlus™ containing *Lactobacillus acidophilus* and *Bifidobacterium* sp., 113 g/day) for 15 days	Non-probiotic yogurt	Less vaginal fungal colonization among women was observed when the women consumed probiotic yogurts; significant effect was noted for DanActive™ (*p* = 0.03)	While in the intervention phase; lower oral fungal colonization observed in HIV(+) women, significantly fewer women used OTC^b^ medication and suffered from constipation	“Consumption of a probiotic yogurt could reduce fungal colonization and some symptoms in HIV-infected and HIV-uninfected women”
Laue et al. (18)	Double blind, placebo-controlled, randomized clinical pilot trial with two parallel arms	BV	36 women aged 36 ± 12 diagnosed with BV; 17/18 women completed *verum* and 17/18 women completed placebo arms of study; 1 woman who completed the *verum* arm halfway into the trial was included in the assessment of secondary parameters (i.e., 33/34 were included in the full assessment; 34/34 women were included in the evaluation of secondary outcomes)	Standard antibiotic (metronidazole, 7 days) and 125 g of yogurt drink produced with *S. thermophilus* and *L. delbrueckii* subsp. *bulgaricus*, containing *L. crispatus*, *L. gasseri*, *L. rhamnosus*, *L. jensenii*, (10^7^ CFU/mL each) for 4 weeks	Standard antibiotic (metronidazole, 7 days) and 125 g chemically acidified milk without bacterial strains for 4 weeks	All (16/16) women were BV-free based on Nugent criteria in *verum* group compared to 13/17 BV-free in placebo group (*p* = 0.103), Nugent score was somewhat lower in *verum* (2.44 ± 1.71) compared to placebo (3.82 ± 3.57) (*p* = 0.444)	All women (17/17) were BV-free in *verum* group based on Amsel criteria, whereas only 11/17 became BV-free in the placebo group. After 4 weeks intervention Amsel score differed (*p* = 0.006) between *verum* (1.18 ± 0.39) and placebo (1.71 ± 1.83) Vaginal pH decreased in the *verum* group compared to the placebo group (*p* = 0.109)	“Additional intake of yoghurt containing *Lactobacillus crispatus* LbV 88 (DSM 22566), *Lactobacillus gasseri* LbV 150 N (DSM 22583), *Lactobacillus jensenii* LbV 116 (DSM 22567) and *Lactobacillus rhamnosus* LbV96 (DSM 22560) probiotic strains improved the recovery rate and symptoms of BV and tended to improve the vaginal microbial pattern”

a Bacterial vaginosis (BV), Vulvovaginal candidiasis (VVC), colony forming units (CFU).

b Over the counter (OTC).

c Premature rupture of membranes (PROM) is a rupture (breaking open) of the membranes (amniotic sac) before labor begins.

#### 3.2.2 Results of observational studies

A total of 3 observational studies exploring the effect of fermented foods for BV/VVC outcomes were identified from the systematic search (Table 2). These studies were generally based on assessment of yogurt consumption patterns (among other factors) and vaginitis outcomes. All three papers reported positive effects related to yogurt consumption. To be more specific, Novikova and Mårdh (24) found that the VVC positive cohort had lower yogurt consumption pattern compared to their VVC negative and control cohorts. The use of certain antibiotics (for non-gynecologic intervention) can disrupt the balance of vaginal microbiota and lead to conditions such as yeast infections or bacterial vaginosis, namely post antibiotic vaginitis (PAV). Pirotta et al. (25) addressed the association between antibiotic use and vaginitis outcomes and reported that some women tend to self-medicate themselves by consuming yogurt and/or probiotic supplements containing lactobacilli. In this cross-sectional study approximately 40% of women resorted to this intervention for prevention and 43% for treatment. In the more recent study by Rosen et al. (26), it was implicated that higher consumption of low-fat dairy (a category of food that inherently may include fermented food such as yogurt, kefir etc.) could confer a healthier microbiome. The study also highlighted a significant scientific gap in understanding the mechanisms linking diet and vaginal microbiota composition.

**Table 2:** Observational studies investigating the effect of ingestion of fermented food on bacterial vaginosis (BV) and vulvovaginal candidiasis (VVC/RVVC).

References	Study design	Disorder^a^	Population	Data collected	Outcome
Novikova and Mårdh (24)	Cohort study	RVVC	83 women with history of VVC divided into two groups (Candida culture-positive, *n* = 43, mean age 26.1 years and Candida culture-negative, *n* = 40, mean age 25.3 years) and Control (women without any history of VVC, *n* = 136)	Assessment of candida status and history via examination and structured questionnaire (1/32 of the etiological factors in the questionnaire was regarding yogurt consumption); Candida culture tests and pH determination was performed	Two factors differed between the groups one of which was yogurt intake. In VCC positive group 28/43 (68%), in VCC negative group 38/40 (95%) and 128/136 (94%) of women in control group regularly consumed yogurt
Pirotta et al. (25)	Cross-sectional survey	VVC and PAV	1,117 women aged 39.5 ± 13 years were included, 798 of which reported VVC history	Written questionnaire where VVC (“thrush”) was defined as vaginal itch, irritation and/or discharge, and PAV as these symptoms occurring within 1 month of taking antibiotics. Survey consisted of 4 sections: lifetime experience of VVC; experience of and risk factors for PAV/VVC in the previous month; self-management of PAV; and demographic information	Yogurt and lactobacillus containing products were the second most prevalent intervention sought by respondents for prevention of PAV. Of the respondents 298/751 (40%) used these products (36% percent orally) for prevention. For treatment, 300/705 (%43) respondents used the products (38% orally). No distinction was made between yogurt and probiotic supplements
Rosen et al. (26)	Cohort/cross-sectional study	BV	634 pregnant women ages 26.1 ± 6 between 26- and 29-weeks gestation	Women completed a self-administered Block food frequency questionnaire (FFQ)	Women in the *L. crispatus* vagitype reported more servings of low-fat dairy, yogurt than women in the other two vagitypes. Women in the *L. crispatus* vagitype reported a median consumption of 9.4 g/day, as compared to 0 g/day in the *L. iners* and BV-mix vagitypes. Yogurt intake was associated with the more favorable *L. crispatus* vagitype

a BV, Bacterial vaginosis; VVC, Vulvovaginal candidiasis; RVVC, recurrent VVC; PAV, Post antibiotic vulvovaginitis.

#### 3.2.3 Quality and bias assessment of interventional and observational studies

**Figure 2:**
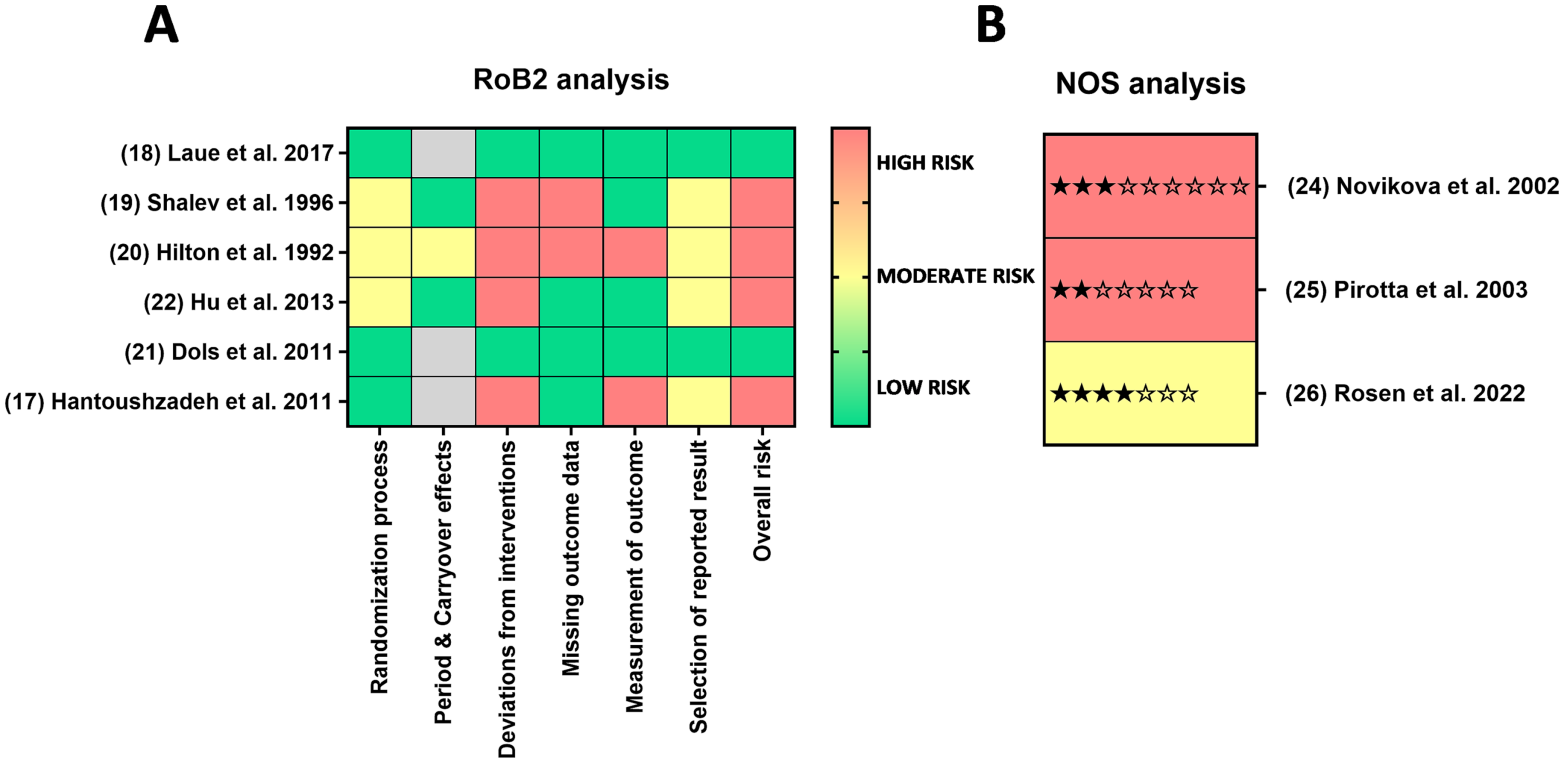
Risk of bias and quality of evidence. Risk of bias was assessed using **(A)** RoB2 for RCT’s and **(B)** NOS analysis for observational studies, categorizing studies into low (green), moderate (yellow), and high (red) risk.

The quality and risk of bias assessments (RoB 2.0 for RCTs and NOS for observational studies) are summarized in Figure 2. Among the six interventional studies assessed using the RoB 2.0 tool, only two were categorized as having a low overall risk of bias, while the remaining four exhibited high risk, most notably in domains such as deviations from intended interventions (*n* = 4), missing outcome data (*n* = 2), and measurement of outcomes (*n* = 2). Additionally, three studies showed concerns regarding selective reporting, randomization errors, or carryover effects. Moderate risks also arose from selective reporting (*n* = 4), errors in the randomization process (*n* = 3), and period or carryover effects in one crossover study. Furthermore, there was a high rate of participant drop-outs in two studies due to refusal to discontinue effective interventions (e.g., yoghurt) (20) or difficulties in adhering to study protocols (19). Observational studies, assessed via the NOS, scored lower overall: Rosen et al. (26) was rated as moderate quality (4/7 stars), while Novikova and Mårdh (24) and Pirotta et al. (25) were rated poor (3/9 and 2/7 stars, respectively), with consistent weaknesses in sample representativeness and lack of detailed comparator information.

These findings suggest that the evidence base is currently stronger for interventional studies, both in quantity (*n* = 6 vs. *n* = 3) and in methodological quality. For example, Laue et al. (18), one of the few low-risk RCTs, provided structured data with both Nugent and Amsel scoring systems, supporting a positive treatment effect. By contrast, the observational studies not only had limited statistical power but also relied heavily on self-reported data and lacked control for confounding factors. This undermines their capacity to establish causality and limits their robustness.

It is also important to acknowledge the heterogeneity in study designs and populations. Sample sizes varied widely (from <20 to 300), populations ranged from pregnant women to HIV-positive individuals, and treatment durations spanned from 1 week to several months. Such variability likely contributes to inconsistent findings and limits the generalizability of results. Furthermore, while all identified studies reported positive outcomes, no neutral or negative findings were captured. This may suggest that studies showing no/negative effect may remain underreported or unpublished. In any case, participant awareness of their assigned intervention was a major contributor to the high risk of bias identified in multiple clinical trials.

Taken together, the predominance of high-risk or poorly rated studies, small sample sizes, and absence of negative data highlight the need for cautious interpretation. While some RCTs, such as Laue et al. (18) and Dols et al. (21), present encouraging results with relatively low bias, the overall certainty of evidence remains moderate at best. Well-powered, rigorously designed trials with transparent reporting and inclusion of negative or neutral outcomes are urgently needed to better ascertain the true effect of fermented foods on vaginal health outcomes.

#### 3.2.4 Characteristics of the fermented foods

Yogurt is a fermented food produced by culturing certain types of dairy ingredients with a bacterial culture that includes *Lactobacillus delbrueckii* subsp. *bulgaricus* and *Streptococcus thermophilus* (27). These two species are not autochthonously present in the human gastrointestinal tract and are not inherently probiotic (28) although specific strains of these species with probiotic capacity have been identified (29). According to a joint report of the Food and Agriculture Organization and World Health Organization (30) probiotics are defined as ‘live microorganisms, which when administered in adequate amounts confer a health benefit on the host’. In order for a product to have probiotic quality it is expected that the probiotic strains retain viability throughout transit of the gastrointestinal tract. Therefore, although standard yogurt may not be probiotic, addition of probiotic cultures prior/post fermentation renders yogurt and other fermented milk products (i.e., milks fermented with starters other than yogurt cultures) carriers for probiotics. In all 9 (interventional and observational) human studies (17–22, 24–26), the food of interest was yogurt or a version thereof (yogurt drink) containing viable microbial load of lactobacilli. Intervention in all RCT studies involved the ingestion of yogurt fermented with standard yogurt culture (*S. thermophilus* and *L. delbrueckii* subsp. *bulgaricus*) and reformulated to include various lactic acid-producing bacteria. Fermented dairy drinks and yogurt are well known sources of lactic acid bacteria (LAB) and the presence of LAB strains are well documented. Indeed, dairy foods (specifically yogurt) are indicated as the carrier of choice for probiotic organisms (31–33) and it is thus reasonable that the clinical trials have explored effects of yogurt consumption. Although LAB are used extensively as culture organisms in dairy foods they are not exclusive to this category and many fermented foods such as fermented cereal drinks and vegetable/fruit juices can be produced through lactic fermentation (31). It is well known that many non-yogurt dairy foods and non-dairy foods may harbor LAB produced via autochthonous lactobacilli or with strains added at the start of fermentation. These may constitute part of the women’s diet. Interestingly, these have not been evaluated as confounders in human studies nor have they been investigated thus far for their efficacy in intervention against or prevention of BV/VVC. Nor have the standard cultures used for fermentation of yogurt in the RCT studies been accounted for or considered as a confounder.

#### 3.2.5 Mechanism of action

The explanations on the mechanism of action centered on the identification of the active component followed by discussions related to how the active component results in the beneficial effects. Three domains were addressed; (1) the translocation mechanism of the probiotics, (2) the effects resulting from colonization of the gut, and (3) mechanisms related to temporal presence of the beneficial microbes in the vaginal econiche.

In all studies, the active component implicated in conferring the beneficial effect is the viable microbial load contained within fermented milks administered/ingested. Several mechanisms explaining how the microorganisms contained in the food matrices may impact the vaginal microenvironment have been proposed. The earliest identified mechanism was the translocation by route of anal contamination and the ascending of bacteria into the vagina (18, 34). This indicates to the rectal microbiota as a reservoir for colonization of the vaginal econiche, evidenced at the strain level for *L. crispatus*, *L. gasseri*, *L. jensenii*, and *L. iners* (35). Moreover, recent publications point to other mechanisms of translocation involving active transport of the bacterial cells. Miller refers to the hematogenous route of bacterial transfer from the gut (36) while other publications center on the cross-talk between the gut and women’s reproductive tract (37). Indeed, the translocation of lactobacilli from the gut of the nursing mother to the mammary glands and expression of the probiotic cells in mother’s milk (38) and the presence of probiotic DNA in the meconium (36) also suggest that there may be more complex, poorly understood translocation mechanisms involved. The involvement of immune system has received attention with possibilities of more directed cellular translocation (39, 40). It has also been proposed that IgA induced regulation for lactobacilli in the small intestine may promote colonization of these bacteria in the vagina (41). It should be stressed here that gut microbiota can also serve as an extravaginal reservoir of BV-associated bacteria (42), therefore, the properly balanced intestinal microbiota and healthy gut epithelium can help maintain a healthy vaginal environment.

The intestinal microbiota is modulated by ingested microorganisms and impacts the host immune system. As shown in the mouse model of *Gardnerella vaginalis* (GV)-induced BV, oral administration of *L. rhamnosus* HN001 and/or *L. acidophilus* GLa-14 more effectively activated innate and adaptive immunity compared to the intravaginal administration (43). Oral administration of lactobacilli more potently inhibited GV-induced myeloperoxidase activity, NF-κB activation, and TNF-α and IL-1β expression (involved in innate immunity), as well as inhibited GV-induced expression of RORγt, TNF-α, and IL-17 (involved in adaptive immunity). These results suggest that the anti-BV effect of orally administered probiotics may be due to its regulatory effects on immune responses through the gastrointestinal tract (43).

The newest proposed mechanism of action by which gut bacteria can beneficially influence the vaginal health involves extracellular vesicles (EV) released by bacteria. EV are small structures (below 300 nm) made of bilayer lipid membranes that cannot replicate themselves but carry a cargo of proteins, nucleic acids, and lipids. They play a key role in immune function, inflammatory reaction, and disease development by transporting active molecules to distant sites through the bloodstream (44). It has been suggested that EVs from commensal bacteria may have beneficial effects on the host by enhancing their mucosal tolerance and preventing disease progression, whereas EVs from pathogenic bacteria have proinflammatory effects on the host immune cells (44). While gut microbiota is restricted to the intestinal lumen, the secreted EVs can penetrate through the intestinal barriers, enter the systemic circulation, and affect both adjacent and distant organs (44). The potential of EVs in mediating lactobacilli beneficial effects was explored in *in vitro* studies in HeLa cervical cells model and showed that EVs from *L. crispatus* BC5 and *L. gasseri* BC12 (isolated from vagina of healthy women) significantly enhanced the cellular adhesion of other vaginal beneficial lactobacilli (45). The same EVs reduced the adhesion of pathogens: *Escherichia coli*, *Staphylococcus aureus*, *S. agalactiae*, and *Enterococcus faecalis* supporting the hypothesis that extracellular vesicles released by symbiotic lactobacilli may be implicated in sustaining a healthy vaginal homeostasis (45). Pili on the cell surface of *Lacticaseibacillus rhamnosus* GG (LGG) promotes adhesion to the mucosa and ensure close contact to host cells and emit EV carrying cargo of effector molecules. These molecules, including secreted proteins, surface-anchored proteins, polysaccharides, and lipoteichoic acids, which interact with host physiological processes have been identified and shown to stimulate epithelial cell survival and integrity, reduce oxidative stress, mitigate excessive mucosal inflammation, enhance IgA secretion, and provide long-term protection through epigenetic imprinting (46).

Temporal presence of lactobacilli in the vaginal epithelium can act protectively by competing with pathogens for nutrients and for adhesion sites at the surface of epithelial cells, producing of hydrogen peroxide, bacteriocins, and biosurfactants, along with organic acids (lactic acid, formic acid and other short chain fatty acids), which maintain the pH of the vagina too low for the growth of pathogens or by modulating local or regional immunological responses (47, 48). These postbiotic molecules could be considered effective against BV as well as VVC. On the other hand, *Candida* yeast morphogenesis and subsequent pathogenesis directed with quorum sensing activity may be disrupted with anti-film forming activity of probiotic enzymes (such as chitinases) and other postbiotics (49, 50). Intestinal colonization with bacteria can antagonize *C. albicans* by reshaping the metabolic environment, forcing metabolic adaptations that reduce fungal pathogenicity (51). Therefore, distinct mechanisms related to enrichment of lactobacilli in the vaginal microbiota can be beneficial for prevention and treatment of both BV and VVC.

#### 3.2.6 Safety assessment

*S. thermophilus* and *L. delbrueckii* spp. *bulgaricus* are generally recognized as safe (GRAS) microorganisms used in the production of yogurt and various dairy products (52). Some specific strains of these species have been studied for their probiotic efficacy (27) however some criteria need addressing for justification in using the term “probiotic” to describe such strains, e.g., the organism must be identified at the strain level and shown to express the relevant trait. Safety is a prerequisite for strains that have been identified as probiotic (53). Probiotic occurrence as normal commensals of the mammalian microbiota and their established safe use in diverse food and supplement products worldwide support their safety for oral consumption. Nevertheless, they are viable organisms, and therefore it is feasible that they could infect the host. Precaution is advised in the administration of probiotic organisms to some populations (i.e., immunocompromised patients) (54). Specific assessment of the probiotic strain provides a more in-depth understanding toward the safety of oral consumption. This has been demonstrated for individual strains such as *L. crispatus* CTV-05 (55) and *L. rhamnosus* HN001 (56). Furthermore, it is important to state that all species included in this review have received a qualified presumption of safety (QPS) status by the European Food Safety Authority (EFSA) (57). FDA regulations indicate the GRAS status of yogurt bacteria and specify granting of permission for the use of harmless lactic acid-producing bacteria, such as *Lactobacillus acidophilus*, as optional ingredients in specified standardized foods (58).

### 3.3 Efficacious probiotic strains and their potential as fermentation organisms

The search for efficacious probiotic strains used as intervention in human clinical trials yielded 56 full-text articles (Figure 1b, Phase IIa) referencing 54 probiotic strains with identifiers. These strains are listed (Supplementary section S2.3) along with the results of their utilization in the Phase IIb search. Of the 54 strains, the majority of the strains (74%) yielded no results (i.e., these strains were not used as fermentation organisms). It was beyond the scope of the present review to present a conclusive evaluation of the efficacy of probiotic supplements for prevention and treatment of BV/VVC. Reviews are available that summarize the most current body of evidence and highlighting the importance of ongoing endeavors for locating efficacious strains (59–61).

The 14 species that are mentioned in food science and technology studies (Supplementary section S2.3), without consideration of their utilization as starters in fermented foods, were strains belonging to the genera *Lacticaseibacillus*, *Lactiplantibacillus*, *Lactobacillus*, *Ligilactobacillus*, and *Limosilactobacillus*, which were previously classified within the broad *Lactobacillus* genus prior to its taxonomic reclassification in 2020. However, after full text assessment and elimination, 9 strains (member to 6 species) were determined to have been investigated for their potential as fermentation organisms in a total of 120 food science and technology articles. These strains and the food categories in which they were investigated are summarized in Table 3. A more detailed table including fermentation conditions, initial and final microbial counts are presented in the Supplementary section S3.3. While strain-level efficacy of specific *Lactobacillus* and *Bifidobacterium* species has been demonstrated in controlled clinical trials, their functional stability and survival within complex food matrices may differ substantially. This distinction is critical to understanding the translational potential of using fermented foods as vehicles for delivering clinically efficacious probiotic strains. For this reason, final viable counts of the strains in fermented foods were evaluated in the following section to address this potential.

**Table 3:** Efficacious probiotic bacteria that have been utilized in food fermentation as pure or co-culture starter strains.

Probiotic strain and identifier	Fermented food origin	Articles	References
*Lacticaseibacillus rhamnosus* (*Lactobacillus rhamnosus*) GG	Animal	18	(62, 63, 67–83)
	Plant	19	(84–102)
	Mixed	11	(64, 102–110)
*Lacticaseibacillus rhamnosus* (*Lactobacillus rhamnosus*) HN001	Animal	8	(111–118)
	Plant	2	(66, 119)
	Mixed	3	(116–118)
*Lacticaseibacillus rhamnosus* (*Lactobacillus rhamnosus*) GR-1	Animal	2	(120, 121)
	Plant	1	(122)
	Mixed	2	(123, 124)
*Lacticaseibacillus rhamnosus* (*Lactobacillus rhamnosus*) IMC 501	Animal	1	(125)
	Mixed	1	(126)
*Lactobacillus acidophilus* LA-5	Animal	40	(83, 111, 127–165)
	Plant	8	(140, 166–173)
	Mixed	11	(65, 109, 110, 164, 165, 174–179)
*Limosilactobacillus reuteri* (*Lactobacillus reuteri*) RC-14	Animal	2	(120, 121)
*Lactobacillus casei rhamnosus* LCR35	Animal	2	(128, 129)
*Lactiplantibacillus plantarum* (*Lactobacillus plantarum*) LP115	Plant	1	(180)
*Lacticaseibacillus paracasei* (*Lactobacillus paracasei*) IMC 502	Animal	2	(125, 181)
	Mixed	1	(126)

Only 9 of the 54 strains identified from the previous systematic search efficacious against BV/VVC were utilized in food fermentations. Most of the 120 studies (87%) utilized either LGG or *Lactobacillus acidophilus* LA-5 (LA-5) as single strain or in co-culture with other starters. Most of the studies (55%) involved utilization of material of animal origin, predominantly dairy, while approximately17% of the studies were performed using plant-based material. Of the studies categorized as animal-based products, only two were concerned with LGG use in fermented meat (62, 63). There were also studies investigating mixed material matrices utilizing plant-based raw materials such as cereals and legumes along with dairy or even insects (64, 65). Aside from yogurt and fermented milk, dairy matrices included cheese. In fermentation of milk and yogurt, counts of probiotic bacteria were generally shown to increase significantly. However, fermentation of cheese products involves ripening the solid material in controlled chambers or submerged in brine. In this process the material is held at refrigeration temperatures for prolonged periods of time. Even for these products, the probiotic viability was retained or increased. Many of the foods were shown to contain up to 6–9 log CFU/g or CFU/mL of the inoculated strains in the final product (Supplementary table S3.3). Unless heat treatment is applied to the food prior to consumption, such as roasting of coffee beans (66), the viability of the probiotics may be preserved to achieve their bioactive potential. It is worth mentioning that RCT’s detailed in Table 1 involved intervention is with yogurt and only the article by Dols et al. (21) specifies a strain that is recaptured in probiotic efficacy studies (namely *L. rhamnosus* GR-1). However, fermented foods studied reflect a wide scope of foods of plant and/or animal origin beyond yogurt. This indicates a distinct research gap for delivery of the probiotics utilizing different food matrices as efficacious agents against BV/VVC. These constitute understudied interventions that should be considered when designing clinical research to investigate this potential.

Several of the strains listed in Table 3 are available as commercial starter cultures. While some products contain single-strain formulations (such as LGG^®^ by Ch. Hansen and HN001 by Danisco), others (such as SYNBIO^®^ and ABT^®^) include multiple probiotic strains. Findings indicate that the availability of the strains for food studies and manufacturing operations may be an important factor enabling some strains to be more intensely investigated for their fermentation starter potential. The specific screening undertaken in Phase II of this review underpins the potential of fermented foods as medium for growth and as vehicles for delivery of probiotic organisms. Furthermore, it is clear that the products that could be utilized in future studies, either for food science and technology research or for clinical research, is not limited to yogurt or fermented milks. Indeed, fermented food has a vast and dynamic scope, evolving as traditional fermented foods are revived and as novel food matrices such as alternative protein sources emerge. Furthermore, it is important to consider that probiotic presence in the food matrix must be investigated to account for metabolites (such as short chain fatty acids), bioactive molecules (such as exopolysaccharides and bioactive peptides) and parabiotic factors (cell wall fragments) which may enhance the therapeutic potential of the fermented foods.

## 4 Conclusion

Human studies demonstrating the efficacy of fermented foods against BV and VVC remain limited. Notably, existing studies have exclusively focused on fermented dairy products (primarily yogurt) where probiotic bacteria serve as the active component. In contrast, a substantial body of clinical evidence supports the effectiveness of probiotic supplements in preventing and treating BV/VVC, among other health conditions. The widespread commercial availability and global distribution of specific strains (such as LGG and LA-5) have likely contributed to their prominence in food fermentation research. However, for many clinically relevant probiotic strains, studies exploring their use as fermentation starters are scarce or nonexistent. Existing literature does suggest a wide variety of potentially suitable fermented foods, including those of animal, plant, or mixed origin, that could serve as vehicles for probiotic delivery in future clinical interventions. Together, these observations highlight a significant opportunity for future research at the intersection of clinical nutrition and food fermentation, aimed at broadening both the diversity of probiotic-containing foods and their therapeutic applications. Future clinical studies planned to assess efficacy of fermented foods should consider that clinical translations may be complex due to the inherent variability of food matrices and fermentation processes, regulatory classification (food vs. therapeutic), strain patentability issues. Thus, it can be highly recommended that these studies are planned in an interdisciplinary arena with the contribution of food scientists and nutritionist. Network initiatives such as COST actions may be useful tools to establish such collaborative efforts, much needed for establishing standardized criteria for selecting, characterizing, and validating probiotic strains for fermented foods targeting vaginal health.

## Data Availability

The original contributions presented in the study are included in the article/Supplementary material, further inquiries can be directed to the corresponding author.
