# Supplementary material for: Efficacy of fermented foods for the prevention and treatment of bacterial vaginosis and vulvovaginal candidiasis
Source: Front Nutr. 2025 Nov 21;12:1658988. doi: 10.3389/fnut.2025.1658988 (PMC12679833; doi:10.3389/fnut.2025.1658988)
Supplement: Supplementary file 1 [file Datasheet_1.docx]

**Supplementary File**

**S.1.1 Search strings adapted from PIMENTO for Section 2.1** (Phase I: Human studies for investigating efficacy of fermented foods against BV and VVC) (functional strings relevant to present study are indicated in red)

|  | **PubMed Database** |
| --- | --- |
|  | Query |
| #1 | "Fermented Foods"[Mesh] OR "Fermentation"[Mesh] OR ("Food"[Mesh] AND ferment*[tiab]) OR ((ferment*[tiab] OR cultur*[tiab] OR leaven*[tiab]) AND (food*[tiab] OR drink*[tiab] OR beverage*[tiab])) OR "Fermented product"[tiab:~6] OR "Fermented products"[tiab:~6] OR "cultured product"[tiab:~6] OR "cultured products"[tiab:~6] OR "product fermentation"[tiab:~6] OR "products fermentation"[tiab:~6] OR "starter culture*"[tiab] OR ((ferment*[tiab] OR culture*[tiab] OR sour*[tiab]) AND (milk[tiab] OR dairy[tiab])) OR buttermilk[tiab] OR sour cream*[tiab] OR cheese*[tiab] OR yoghurt[tiab] OR yogurt[tiab] OR "yoghourt"[tiab] OR "yakult"[tiab] OR "quark"[tiab] OR "kefir"[tiab] OR "lassi"[tiab] OR "kumis"[tiab] OR "koumiss"[tiab] OR "kajmak"[tiab] OR "airag"[tiab] OR "ayran"[tiab] OR "calpis"[tiab] OR "borhani"[tiab] OR "chal"[tiab] OR "doogh"[tiab] OR kvass[tiab] OR skyr[tiab] OR amasi[tiab] OR bouza[tiab] OR butter*[tiab] OR chal[tiab] OR filmjolk[tiab] OR kishk[tiab] OR labne*[tiab] OR ((Ferment*[tiab] OR cultur*[tiab] OR cured[tiab]) AND (meat*[tiab] OR fish*[tiab] OR seafood*[tiab] OR shellfish[tiab] OR sausage*[tiab])) OR "salami"[tiab] OR "pepperoni"[tiab] OR peperoni[tiab] OR "chorizo"[tiab] OR "cervelat"[tiab] OR "mettwurst"[tiab] OR "summer sausage"[tiab] OR "sucuk"[tiab] OR "dried meat"[tiab] OR "dried sausage"[tiab] OR "dry sausage"[tiab] OR "fish sauce"[tiab] OR "shrimp paste"[tiab] OR "shrimp sauce"[tiab] OR "oyster sauce"[tiab] OR "prosciutto"[tiab] OR "pancetta"[tiab] OR "saucisson"[tiab] OR sucuk[tiab] OR ((Ferment*[tiab] OR cultur*[tiab]) AND (fruit*[tiab] OR vegetable*[tiab] OR coconut*[tiab] OR almond*[tiab] OR hazelnut*[tiab] OR nut[tiab] OR cucumber*[tiab] OR lemon*[tiab] OR citrus[tiab] OR cabbage*[tiab] OR cauliflower*[tiab] OR pepper*[tiab] OR carrot*[tiab] OR olive*[tiab] OR onion*[tiab] OR sago[tiab])) OR "sauerkraut"[tiab] OR "table olive"[tiab] OR pickle*[tiab] OR "kimchi"[tiab] OR "paocai"[tiab] OR torshi[tiab] OR ((Ferment*[tiab] OR cultur*[tiab]) AND (tea[tiab] OR teas[tiab] OR juice*[tiab])) OR "beer"[tiab] OR "wine"[tiab] OR "cider*"[tiab] OR shochu[tiab] OR "kombucha*"[tiab] OR "pulque"[tiab] OR puer[tiab] OR pu’er[tiab] OR pu-er[tiab] OR pu-erh[tiab] OR "pu erh"[tiab] OR "fuzhuan"[tiab] OR "dark tea*"[tiab] OR "yellow tea*"[tiab] OR coffee[tiab] OR shalgam[tiab] OR hardaliye[tiab] OR ((Ferment*[tiab] OR cultur*[tiab]) AND (soy[tiab] OR soya[tiab] OR bean*[tiab] OR pea[tiab] OR peas[tiab] OR lentil*[tiab] OR chickpea*[tiab] OR legume*[tiab] OR pulse*[tiab] OR (poi[tiab])) OR "soy sauce"[tiab] OR "soya sauce"[tiab] OR "soybean paste"[tiab] OR "miso"[tiab] OR "tempeh"[tiab] OR tempe[tiab] OR "natto"[tiab] OR "doenjang"[tiab] OR "doubanjiang"[tiab] OR douchi[tiab] OR "gochujang"[tiab] OR cheonggukjang[tiab] OR tsukemono[tiab] OR garri[tiab] OR ((Ferment*[tiab] OR cultur*[tiab] OR leaven*[tiab]) AND (cereal*[tiab] OR grain*[tiab] OR wheat*[tiab] OR oat[tiab] OR oats[tiab] OR rice*[tiab] OR millet*[tiab] OR sorghum*[tiab] OR maize*[tiab] OR rye[tiab] OR barley*[tiab] OR chia[tiab] OR oilseed*[tiab] OR teff[tiab])) OR "bread"[tiab] OR "sourdough"[tiab] OR "crispbread"[tiab] OR "boza"[tiab] OR "ogi"[tiab] OR dosa[tiab] OR "tarhana"[tiab] OR "buckwheat"[tiab] OR "spelt"[tiab] OR "einkorn"[tiab] OR "quinoa"[tiab] OR "amaranth"[tiab] OR "tef"[tiab] OR "bushera"[tiab] OR chica[tiab] OR chicha[tiab] OR choujiu[tiab] OR injera[tiab] OR mahewu[tiab] OR ogiri[tiab] OR pozol[tiab] OR ugba[tiab]) OR ((Ferment*[tiab] OR cultur*[tiab]) AND ("condiment*"[tiab] OR relish*[tiab] OR horseradish[tiab] OR "dressing*"[tiab] OR "seasoning*"[tiab] OR "sauce*"[tiab] OR cocoa*[tiab] OR tuber[tiab] OR "acetic acid"[tiab])) OR "chocolate*"[tiab] OR "vinegar*"[tiab] OR "tabasco"[tiab] OR "sriracha"[tiab] OR "Worcestershire"[tiab] OR "Worcester"[tiab] |
| #2 | "Diet"[Mesh] OR "Life Style"[Mesh] OR "Eating"[Mesh] OR "Feeding Behavior"[Mesh] OR ((food[tiab] OR macronutrient*[tiab] OR eating[tiab]) AND (intake*[tiab] OR habit*[tiab] OR behavior*[tiab] OR pattern*[tiab])) OR diet*[tiab] OR intake[tiab] OR ingestion[tiab] OR suppl*[tiab] OR consumption[tiab] OR meal*[tiab] OR nutrient*[tiab] OR nutrit*[tiab] |
| #3 | (vaginit* OR vaginos* OR candidos* OR candidi* OR thrush OR ((vagin*) AND candida) OR monili* OR vulvovagin* OR “vaginal infection” OR “vaginal yeast infection” OR “vaginal yeast infections”) |
| #4 | #1 AND #2 AND #3 |
| #5 | "Diet Surveys"[Mesh] OR "Cohort Studies"[Mesh] OR cohort*[Tiab] OR prospective[Tiab] OR longitudinal[Tiab] |
| #6 | Randomized Controlled Trial[Publication Type] OR Controlled Clinical Trial[Publication Type] OR Pragmatic Clinical Trial[Publication Type] OR Clinical Study[Publication Type] OR Adaptive Clinical Trial[Publication Type] OR Equivalence Trial[Publication Type] OR Clinical Trial[Publication Type] OR Clinical Trial, Phase I[Publication Type] OR Clinical Trial, Phase II[Publication Type] OR Clinical Trial, Phase III[Publication Type] OR Clinical Trial, Phase IV[Publication Type] OR Clinical Trial Protocol[Publication Type] OR multicenter study[Publication Type] OR "Clinical Studies as Topic"[Mesh] OR "Clinical Trials as Topic"[Mesh] OR "Clinical Trial Protocols as Topic"[Mesh] OR "Multicenter Studies as Topic"[Mesh] OR "Random Allocation"[Mesh] OR "Double-Blind Method"[Mesh] OR "Single-Blind Method"[Mesh] OR "Placebos"[Mesh:NoExp] OR "Control Groups"[Mesh] OR "Cross-Over Studies"[Mesh] OR random*[Title/Abstract] OR sham[Title/Abstract] OR placebo*[Title/Abstract] OR ((singl*[Title/Abstract] OR doubl*[Title/Abstract]) AND (blind*[Title/Abstract] OR dumm*[Title/Abstract] OR mask*[Title/Abstract])) OR ((tripl*[Title/Abstract] OR trebl*[Title/Abstract]) AND (blind*[Title/Abstract] OR dumm*[Title/Abstract] OR mask*[Title/Abstract])) OR "control study"[tiab:~3] OR "control studies"[tiab:~3] OR "control group"[tiab:~3] OR "control groups"[tiab:~3] OR "healthy volunteers"[tiab:~3] OR "control trial"[tiab:~3] OR "control trials"[tiab:~3] OR "controlled study"[tiab:~3] OR "controlled trial"[tiab:~3] OR "controlled studies"[tiab:~3] OR "controlled trials"[tiab:~3] OR "clinical study"[tiab:~3] OR "clinical studies"[tiab:~3] OR "clinical trial"[tiab:~3] OR "clinical trials"[tiab:~3] OR Nonrandom*[Title/Abstract] OR non random*[Title/Abstract] OR non-random*[Title/Abstract] OR quasi-random*[Title/Abstract] OR quasirandom*[Title/Abstract] OR "phase study"[tiab:~3] OR "phase studies"[tiab:~3] OR "phase trial"[tiab:~3] OR "phase trials"[tiab:~3] OR "crossover study"[tiab:~3] OR "crossover studies"[tiab:~3] OR "crossover trial"[tiab:~3] OR "crossover trials"[tiab:~3] OR "cross-over study"[tiab:~3] OR "cross-over studies"[tiab:~3] OR "cross-over trial"[tiab:~3] OR "cross-over trials"[tiab:~3] OR ((multicent*[tiab] OR multi-cent*[tiab] OR open label[tiab] OR open-label[tiab] OR equivalence[tiab] OR superiority[tiab] OR non-inferiority[tiab] OR noninferiority[tiab] OR quasiexperimental[tiab] OR quasi-experimental[tiab]) AND (study[tiab] OR studies[tiab] OR trial*[tiab])) OR allocated[tiab] OR pragmatic study[tiab] OR pragmatic studies[tiab] OR pragmatic trial*[tiab] OR practical trial*[tiab] |
| #7 | "Epidemiologic Methods"[Mesh:NoExp] OR "Epidemiologic Studies"[Mesh] OR "Observational Studies as Topic"[Mesh] OR "Clinical Studies as Topic"[Mesh] OR "Single-Case Studies as Topic"[Mesh] OR "Organizational Case Studies"[Mesh] OR observational study[Publication Type] OR validation study[Publication Type] OR clinical study[Publication Type] OR case reports[Publication Type] OR "observational study"[tiab:~3] OR "observational studies"[tiab:~3] OR "observational design"[tiab:~3] OR "observational analysis"[tiab:~3] OR "observational analyses"[tiab:~3] OR ((cohort*[tiab] OR prospective[tiab] OR follow-up[tiab] OR longitudinal[tiab] OR long-term[tiab] OR retrospective[tiab]) AND (study[tiab] OR studies[tiab] OR design[tiab] OR analysis[tiab] OR analyses[tiab] OR data[tiab] OR review[tiab])) OR case control*[tiab] OR case comparison*[tiab] OR case-referent[tiab] OR "population study"[tiab:~3] OR "population studies"[tiab:~3] OR "population analysis"[tiab:~3] OR "population analyses"[tiab:~3] OR "descriptive study"[tiab:~3] OR "descriptive studies"[tiab:~3] OR "descriptive design"[tiab:~3] OR "descriptive analysis"[tiab:~3] OR "descriptive analyses"[tiab:~3] OR "multidimensional study"[tiab:~3] OR "multidimensional studies"[tiab:~3] OR "multidimensional design"[tiab:~3] OR "multidimensional analysis"[tiab:~3] OR "multidimensional analyses"[tiab:~3] OR "cross-sectional study"[tiab:~3] OR "cross-sectional studies"[tiab:~3] OR "cross-sectional design"[tiab:~3] OR "cross-sectional analysis"[tiab:~3] OR "cross-sectional analyses"[tiab:~3] OR "cross-sectional research"[tiab:~3] OR "cross-sectional survey"[tiab:~3] OR "cross-sectional findings"[tiab:~3] OR natural experiment*[tiab] OR quasi experiment*[tiab] OR "nonexperimental study"[tiab:~3] OR "nonexperimental studies"[tiab:~3] OR "nonexperimental design"[tiab:~3] OR "nonexperimental analysis"[tiab:~3] OR "nonexperimental analyses"[tiab:~3] OR "prevalence study"[tiab:~3] OR "prevalence studies"[tiab:~3] OR "prevalence analysis"[tiab:~3] OR "prevalence analyses"[tiab:~3] OR case series[tiab] OR "case report"[tiab:~3] OR "case reports"[tiab:~3] OR "case study"[tiab:~3] OR "case studies"[tiab:~3] OR "case histories"[tiab:~3] |
| #8 | "systematic review" |
| #9 | #5 OR #6 OR #7 OR #8 |
| #10 | #4 AND #9 |
| #11 | #10 NOT (("Child"[Mesh] OR "Infant"[Mesh] OR "Adolescent"[Mesh]) NOT "Adult"[Mesh]) |
| #12 | #11 NOT (("Animals"[Mesh] OR "Animal Experimentation"[Mesh] OR "Models, Animal"[Mesh] OR "Vertebrates"[Mesh]) NOT ("Humans"[Mesh] OR "Human Experimentation"[Mesh])) |
| #13 | #12 NOT ("Breast Feeding"[Majr] OR "Milk, Human"[Majr]) |
| #14 | #13 AND (English[Filter]) |
|  |  |
|  | **Scopus Database** |
| #1 | TITLE-ABS-KEY ((ferment* OR cultur* OR leaven*) W/6 (food* OR drink* OR beverage*) OR "starter culture*") OR TITLE-ABS-KEY ((ferment* OR cultur* OR leaven*) W/2 product*) OR TITLE-ABS-KEY (((ferment* OR culture* OR sour*) W/6 (milk OR dairy OR cream* OR quark)) OR buttermilk OR cheese* OR yoghurt OR yogurt OR yoghourt OR yakult OR kefir OR lassi OR kumis OR koumiss OR kajmak OR airag OR ayran OR calpis OR borhani OR chal OR doogh OR kvass OR skyr OR amasi OR bouza OR butter* OR chal OR filmjolk OR kishk OR labne*) OR TITLE-ABS-KEY (((Ferment* OR cultur* OR cured) W/6 (meat* OR fish* OR seafood* OR shellfish OR sausage*)) OR "salami" OR "pepperoni" OR peperoni OR "chorizo" OR "cervelat" OR "mettwurst" OR "summer sausage" OR "sucuk" OR "dried meat*" OR "dried sausage*" OR "dry sausage*" OR "fish sauce*" OR "shrimp paste" OR "shrimp sauce" OR "oyster sauce" OR "prosciutto" OR "pancetta" OR "saucisson" OR sucuk) OR TITLE-ABS-KEY (((Ferment* OR cultur*) W/6 (fruit* OR vegetable* OR coconut* OR almond* OR hazelnut* OR nut OR cucumber* OR lemon* OR citrus OR cabbage* OR cauliflower* OR pepper* OR carrot* OR olive* OR onion* OR sago)) OR "sauerkraut" OR "table olive*" OR pickle* OR "kimchi" OR "paocai" OR torshi) OR TITLE-ABS-KEY (((Ferment* OR cultur*) W/6 (tea OR teas OR juice*)) OR "beer" OR "wine" OR cider* OR shochu OR kombucha* OR "pulque" OR puer OR "pu-er*" OR "fuzhuan" OR "dark tea*" OR "yellow tea*" OR coffee OR shalgam OR hardaliye) OR TITLE-ABS-KEY (((Ferment* OR cultur*) W/6 (soy OR soya OR bean* OR pea OR peas OR lentil* OR chickpea* OR legume* OR pulse* OR poi)) OR "soy* sauce*" OR "soybean paste*" OR miso* OR tempeh* OR tempe OR "natto" OR "doenjang" OR "doubanjiang" OR douchi OR "gochujang" OR cheonggukjang OR tsukemono OR garri) OR TITLE-ABS-KEY (((Ferment* OR cultur* OR leaven*) W/6 (cereal* OR grain* OR wheat* OR oat OR oats OR rice* OR millet* OR sorghum* OR maize* OR rye OR barley* OR chia OR oilseed* OR teff)) OR "bread" OR "sourdough" OR "crispbread" OR "boza" OR "ogi" OR dosa OR "tarhana" OR "buckwheat" OR "spelt" OR "einkorn" OR "quinoa" OR "amaranth" OR "tef" OR "bushera" OR chica OR chicha OR choujiu OR injera OR mahewu OR ogiri OR pozol OR ugba) OR TITLE-ABS-KEY (((Ferment* OR cultur*) W/6 (condiment* OR relish* OR horseradish OR dressing* OR seasoning* OR sauce* OR cocoa* OR tuber OR "acetic acid")) OR chocolate* OR vinegar* OR "tabasco" OR "sriracha" OR "Worcestershire" OR "Worcester") |
| #2 | TITLE-ABS-KEY (((food OR *nutrient* OR eating OR nutrit*) W/6 (intake* OR habit* OR behavior* OR pattern* OR consumption OR suppl* OR ingestion)) OR diet* OR meal*) |
| #3 | TITLE-ABS-KEY ((vaginit* OR vaginos* OR candidos* OR candidi* OR thrush OR ((vagin*) AND candida) OR monili* OR vulvovagin* OR "vaginal infection" OR "vaginal yeast infection" OR "vaginal yeast infections")) |
| #4 | #1 AND #2 AND #3 (add combination of string numbers to the field in advanced search in the field "Combined queries…") |
| #5 | TITLE-ABS-KEY (random* OR sham OR placebo*) OR TITLE-ABS-KEY ((singl* OR doubl*) W/1 (blind* OR dumm* OR mask*)) OR TITLE-ABS-KEY ((tripl* OR trebl*) W/1 (blind* OR dumm* OR mask*)) OR TITLE-ABS-KEY (control* W/3 (study OR studies OR trial* OR group*)) OR TITLE-ABS-KEY (clinical W/3 (study OR studies OR trial*)) OR TITLE-ABS-KEY (Nonrandom* OR "non random*" OR non-random* OR quasi-random* OR quasirandom*) OR TITLE-ABS-KEY (phase W/3 (study OR studies OR trial*)) OR TITLE-ABS-KEY ((crossover OR cross-over) W/3 (study OR studies OR trial*)) OR TITLE-ABS-KEY ((multicent* OR multi-cent*) W/3 (study OR studies OR trial*)) OR TITLE-ABS (allocated) OR TITLE-ABS-KEY (("open label" OR open-label) W/5 (study OR studies OR trial*)) OR TITLE-ABS-KEY ((equivalence OR superiority OR non-inferiority OR noninferiority) W/3 (study OR studies OR trial*)) OR TITLE-ABS-KEY ("pragmatic study" OR "pragmatic studies") OR TITLE-ABS-KEY ((pragmatic OR practical) W/3 trial*) OR TITLE-ABS-KEY ((quasiexperimental OR quasi-experimental) W/3 (study OR studies OR trial*)) OR TITLE (trial) OR KEY (trial) |
| #6 | TITLE-ABS-KEY (observational W/3 (study OR studies OR design OR analysis OR analyses)) OR TITLE-ABS-KEY (cohort*) OR TITLE-ABS-KEY (prospective W/7 (study OR studies OR design OR analysis OR analyses)) OR TITLE-ABS-KEY (("follow up" OR followup) W/7 (study OR studies OR design OR analysis OR analyses)) OR TITLE-ABS-KEY ((longitudinal OR longterm OR (long W/1 term)) W/7 (study OR studies OR design OR analysis OR analyses OR data)) OR TITLE-ABS-KEY (retrospective W/7 (study OR studies OR design OR analysis OR analyses OR data OR review)) OR TITLE-ABS-KEY ((case W/1 control) OR (case W/1 comparison) OR (case W/1 controlled)) OR TITLE-ABS-KEY (case-referent W/3 (study OR studies OR design OR analysis OR analyses)) OR TITLE-ABS-KEY (population W/3 (study OR studies OR analysis OR analyses)) OR TITLE-ABS-KEY (descriptive W/3 (study OR studies OR design OR analysis OR analyses)) OR TITLE-ABS-KEY ((multidimensional OR (multi W/1 dimensional)) W/3 (study OR studies OR design OR analysis OR analyses)) OR TITLE-ABS-KEY (cross W/1 sectional W/7 (study OR studies OR design OR research OR analysis OR analyses OR survey OR findings)) OR TITLE-ABS-KEY ((natural W/1 experiment) OR (natural W/1 experiments)) OR TITLE-ABS-KEY (quasi W/1 (experiment OR experiments OR experimental)) OR TITLE-ABS-KEY (("non experiment" OR nonexperiment OR "non experimental" OR nonexperimental) W/3 (study OR studies OR design OR analysis OR analyses)) OR TITLE-ABS-KEY (prevalence W/3 (study OR studies OR analysis OR analyses)) OR TITLE-ABS-KEY ("case series") OR TITLE-ABS-KEY (case W/3 (report OR reports OR study OR studies OR histories)) |
| #7 | TITLE-ABS-KEY ("systematic review") |
| #8 | #5 OR #6 OR #7 (add combination of string numbers to the field in advanced search in the field "Combined queries…") |
| #9 | #4 AND #8 (add combination of string numbers to the field in advanced search in the field "Combined queries…") |
| #10 | (KEY (animal* OR nonhuman)) AND NOT (KEY (human*)) |
| #11 | #9 AND NOT #10 (add combination of string numbers to the field in advanced search in the field "Combined queries…") |
| #12 | (KEY (infant* OR child*)) AND NOT (KEY (adult* OR aged)) |
| #13 | #11 AND NOT #12 (add combination of string numbers to the field in advanced search in the field "Combined queries…") |
|  | Limit #13 to English using the language filter |
|  |  |
|  | **Cochrane Library** |
| #1 | ((ferment* OR cultur* OR leaven*) NEAR/6 (food* OR drink* OR beverage* OR product*) OR (starter NEXT culture*)) OR (((ferment* OR culture* OR sour*) NEAR/6 (milk OR dairy OR cream*)) OR buttermilk OR cheese* OR yoghurt OR yogurt OR yoghourt OR yakult OR quark OR kefir OR lassi OR kumis OR koumiss OR kajmak OR airag OR ayran OR calpis OR borhani OR chal OR doogh OR kvass OR skyr OR amasi OR bouza OR butter* OR chal OR filmjolk OR kishk OR labne*) OR (((Ferment* OR cultur* OR cured) NEAR/6 (meat* OR fish* OR seafood* OR shellfish OR sausage*)) OR "salami" OR "pepperoni" OR peperoni OR "chorizo" OR "cervelat" OR "mettwurst" OR "summer sausage" OR "sucuk" OR (dried NEXT meat*) OR (dried NEXT sausage*) OR (dry NEXT sausage*) OR (fish NEXT sauce*) OR "shrimp paste" OR "shrimp sauce" OR "oyster sauce" OR "prosciutto" OR "pancetta" OR "saucisson" OR sucuk) OR (((Ferment* OR cultur*) NEAR/6 (fruit* OR vegetable* OR coconut* OR almond* OR hazelnut* OR nut OR cucumber* OR lemon* OR citrus OR cabbage* OR cauliflower* OR pepper* OR carrot* OR olive* OR onion* OR sago)) OR "sauerkraut" OR (table NEXT olive*) OR pickle* OR "kimchi" OR "paocai" OR torshi) OR (((Ferment* OR cultur*) NEAR/6 (tea OR teas OR juice*)) OR "beer" OR "wine" OR cider* OR shochu OR kombucha* OR "pulque" OR puer OR pu-er* OR "fuzhuan" OR (dark NEXT tea*) OR (yellow NEXT tea*) OR coffee OR shalgam OR hardaliye) OR (((Ferment* OR cultur*) NEAR/6 (soy OR soya OR bean* OR pea OR peas OR lentil* OR chickpea* OR legume* OR pulse* OR poi)) OR (soy* NEXT sauce*) OR (soybean NEXT paste*) OR miso* OR tempeh* OR tempe OR "natto" OR "doenjang" OR "doubanjiang" OR douchi OR "gochujang" OR cheonggukjang OR tsukemono OR garri) OR (((Ferment* OR cultur* OR leaven*) NEAR/6 (cereal* OR grain* OR wheat* OR oat OR oats OR rice* OR millet* OR sorghum* OR maize* OR rye OR barley* OR chia OR oilseed* OR teff)) OR "bread" OR "sourdough" OR "crispbread" OR "boza" OR “ogi” OR dosa OR "tarhana" OR "buckwheat" OR "spelt" OR "einkorn" OR "quinoa" OR "amaranth" OR "tef" OR "bushera" OR chica OR chicha OR choujiu OR injera OR mahewu OR ogiri OR pozol OR ugba) OR (((Ferment* OR cultur*) NEAR/6 (condiment* OR relish* OR horseradish OR dressing* OR seasoning* OR sauce* OR cocoa* OR tuber OR "acetic acid")) OR chocolate* OR vinegar* OR "tabasco" OR "sriracha" OR "Worcestershire" OR "Worcester") |
| #2 | (((food OR macronutrient* OR eating) NEAR/6 (intake* OR habit* OR behavior* OR pattern*)) OR diet* OR intake OR ingestion OR suppl* OR consumption OR meal* OR nutrient* OR nutrit*) |
| #3 | vaginit* OR vaginos* OR candidos* OR candidi* OR thrush OR (vagin* AND candida) OR monili* OR vulvovagin* OR "vaginal infection" OR "vaginal yeast infection" OR "vaginal yeast infections" |
| #4 | #1 AND #2 AND #3 |
|  | Search results limited with filter "Title Abstract Keyword" |
|  | Results from both Cochrane Reviews and Trials were used for data selection |

**S1.2 P/I, O criteria and Study Type for Section 2.1** (Phase I: Human studies for investigating efficacy of fermented foods against BV and VVC)

| **Population** | Inclusion: All female subjects (women) of/after reproductive age (over 13 years of age), including menopausal women, pregnant women, nursing women.  Exclusion: Studies involving female subjects aged under 13 years. |
| --- | --- |
| **Intervention** | The intervention/exposure consists of the ingestion of any of the fermented foods contained in the PIMENTO search string for fermented foods across the following food groups: dairy, meat and fish, fruits and vegetables, beverages, legumes, cereals and grains. Alcoholic beverages with an alcohol content of more than 1.25% will be excluded. Unless specified otherwise, no limits are set for duration or dosage of the ingested fermented food(s). Unless specified otherwise, studies investigating application of fermented foods other than for nutritional purpose (e.g., nasal or topical) will be excluded. In addition, studied investigating probiotics will be excluded unless the probiotics is/are added at the beginning of the fermentation process and that there are indications from the literature that the probiotic strain(s) contribute(s) to the fermentation of the food matrix. Interventions including any possible confounders such as prebiotic fibers or added bioactive compounds will not be included. Intervention could be designed as a stand-alone intervention or as a combined intervention if the comparator conditions are adequately controlled for non-fermented interventions. |
| **Outcome** | Percent of clinical cure, reduced diversity in vaginal microbiome, recolonization with Lactobacilli, improved Nugent score (7-10), decrease in symptoms will constitute the basis for quantitatively evaluating measure of effect and assessment of recovery from vaginosis, restoration of normal vaginal function, microbiome/microflora and/or reduction in risk of recurrence. Other outcomes reported in the studies that are relevant to the medical prognosis of BV and VVC will also be considered. Population studies where the frequency of consumption of fermented foods have been investigated for the effect on prevalence and recurrence of BV and VVC will also be made use of. Such observational (qualitative) studies on human subjects will be assessed based on the information provided in the article text. |
| **Types of Study** | - Publications reporting on human intervention (efficacy) studies (e.g., randomized controlled studies, randomized uncontrolled studies, non-randomized controlled studies, other intervention studies) are included - Publications reporting on human observational studies (e.g., cohort studies, case-control studies, cross-sectional studies, other observational studies) are included - Available systematic reviews with or without meta-analysis are used to check for potentially missing studies - Animal and *in vitro* studies are excluded |

**S2.1 Search strings adapted from PIMENTO for Section 2.2** (Phase IIa: identification of RCT studies on efficacious probiotic supplements against BV and VVC) (functional strings relevant to present study are indicated in red)

| **PubMed Database** **Query** |
| --- |
| (("vaginit*"[Title/Abstract] OR "vaginos*"[Title/Abstract] OR "candidos*"[Title/Abstract] OR "candidi*"[Title/Abstract] OR "thrush"[Title/Abstract] OR ("vagin*"[Title/Abstract] AND "candida"[Title/Abstract]) OR "monili*"[Title/Abstract] OR "vulvovagin*"[Title/Abstract] OR "vaginal infection"[Title/Abstract] OR "vaginal yeast infection"[Title/Abstract] OR "vaginal yeast infections"[Title/Abstract]) AND ("randomized controlled trial"[Publication Type] OR "controlled clinical trial"[Publication Type] OR "pragmatic clinical trial"[Publication Type] OR "clinical study"[Publication Type] OR "adaptive clinical trial"[Publication Type] OR "equivalence trial"[Publication Type] OR "clinical trial"[Publication Type] OR "clinical trial, phase i"[Publication Type] OR "clinical trial, phase ii"[Publication Type] OR "clinical trial, phase iii"[Publication Type] OR "clinical trial, phase iv"[Publication Type] OR "clinical trial protocol"[Publication Type] OR "multicenter study"[Publication Type] OR "Clinical Studies as Topic"[MeSH Terms] OR "Clinical Trials as Topic"[MeSH Terms] OR "Clinical Trial Protocols as Topic"[MeSH Terms] OR "Multicenter Studies as Topic"[MeSH Terms] OR "Random Allocation"[MeSH Terms] OR "Double-Blind Method"[MeSH Terms] OR "Single-Blind Method"[MeSH Terms] OR "Placebos"[MeSH Terms:noexp] OR "Control Groups"[MeSH Terms] OR "cross over studies"[MeSH Terms] OR "random*"[Title/Abstract] OR "sham"[Title/Abstract] OR "placebo*"[Title/Abstract] OR (("singl*"[Title/Abstract] OR "doubl*"[Title/Abstract]) AND ("blind*"[Title/Abstract] OR "dumm*"[Title/Abstract] OR "mask*"[Title/Abstract])) OR (("tripl*"[Title/Abstract] OR "trebl*"[Title/Abstract]) AND ("blind*"[Title/Abstract] OR "dumm*"[Title/Abstract] OR "mask*"[Title/Abstract])) OR "control study"[Title/Abstract:~3] OR "control studies"[Title/Abstract:~3] OR "control group"[Title/Abstract:~3] OR "Control Groups"[Title/Abstract:~3] OR "healthy volunteers"[Title/Abstract:~3] OR "control trial"[Title/Abstract:~3] OR "control trials"[Title/Abstract:~3] OR "controlled study"[Title/Abstract:~3] OR "controlled trial"[Title/Abstract:~3] OR "controlled studies"[Title/Abstract:~3] OR "controlled trials"[Title/Abstract:~3] OR "clinical study"[Title/Abstract:~3] OR "clinical studies"[Title/Abstract:~3] OR "clinical trial"[Title/Abstract:~3] OR "clinical trials"[Title/Abstract:~3] OR "nonrandom*"[Title/Abstract] OR "non random*"[Title/Abstract] OR "non random*"[Title/Abstract] OR "quasi random*"[Title/Abstract] OR "quasirandom*"[Title/Abstract] OR "phase study"[Title/Abstract:~3] OR "phase studies"[Title/Abstract:~3] OR "phase trial"[Title/Abstract:~3] OR "phase trials"[Title/Abstract:~3] OR "crossover study"[Title/Abstract:~3] OR "crossover studies"[Title/Abstract:~3] OR "crossover trial"[Title/Abstract:~3] OR "crossover trials"[Title/Abstract:~3] OR "cross-over study"[Title/Abstract:~3] OR "cross over studies"[Title/Abstract:~3] OR "cross-over trial"[Title/Abstract:~3] OR "cross-over trials"[Title/Abstract:~3] OR (("multicent*"[Title/Abstract] OR "multi cent*"[Title/Abstract] OR "open-label"[Title/Abstract] OR "open-label"[Title/Abstract] OR "Equivalence"[Title/Abstract] OR "superiority"[Title/Abstract] OR "non-inferiority"[Title/Abstract] OR "noninferiority"[Title/Abstract] OR "quasiexperimental"[Title/Abstract] OR "quasi-experimental"[Title/Abstract]) AND ("Study"[Title/Abstract] OR "studies"[Title/Abstract] OR "trial*"[Title/Abstract])) OR "allocated"[Title/Abstract] OR "pragmatic study"[Title/Abstract] OR "pragmatic studies"[Title/Abstract] OR "pragmatic trial*"[Title/Abstract] OR "practical trial*"[Title/Abstract]) AND ("probiotics"[MeSH Terms] OR "probiotic"[Text Word])) AND ((clinicaltrial[Filter] OR randomizedcontrolledtrial[Filter]) AND (english[Filter])) |
|  |
| **Scopus Database Query** |
| ( TITLE-ABS-KEY ( random* OR sham OR placebo* ) OR TITLE-ABS-KEY ( ( singl* OR doubl* ) W/1 ( blind* OR dumm* OR mask* ) ) OR TITLE-ABS-KEY ( ( tripl* OR trebl* ) W/1 ( blind* OR dumm* OR mask* ) ) OR TITLE-ABS-KEY ( control* W/3 ( study OR studies OR trial* OR group* ) ) OR TITLE-ABS-KEY ( clinical W/3 ( study OR studies OR trial* ) ) OR TITLE-ABS-KEY ( nonrandom* OR "non random*" OR non-random* OR quasi-random* OR quasirandom* ) OR TITLE-ABS-KEY ( phase W/3 ( study OR studies OR trial* ) ) OR TITLE-ABS-KEY ( ( crossover OR cross-over ) W/3 ( study OR studies OR trial* ) ) OR TITLE-ABS-KEY ( ( multicent* OR multi-cent* ) W/3 ( study OR studies OR trial* ) ) OR TITLE-ABS ( allocated ) OR TITLE-ABS-KEY ( ( "open label" OR open-label ) W/5 ( study OR studies OR trial* ) ) OR TITLE-ABS-KEY ( ( equivalence OR superiority OR non-inferiority OR noninferiority ) W/3 ( study OR studies OR trial* ) ) OR TITLE-ABS-KEY ( "pragmatic study" OR "pragmatic studies" ) OR TITLE-ABS-KEY ( ( pragmatic OR practical ) W/3 trial* ) OR TITLE-ABS-KEY ( ( quasiexperimental OR quasi-experimental ) W/3 ( study OR studies OR trial* ) ) OR TITLE ( trial ) OR KEY ( trial ) ) AND ( TITLE-ABS-KEY ( probiotic* ) ) AND ( TITLE-ABS-KEY ( ( vaginit* OR vaginos* OR candidos* OR candidi* OR thrush OR ( ( vagin* ) AND candida ) OR monili* OR vulvovagin* OR "vaginal infection" OR "vaginal yeast infection" OR "vaginal yeast infections" ) ) ) AND ( LIMIT-TO ( LANGUAGE , "English" ) ) |
|  |
| **Cochrane Library Query** |
| (vaginit* OR vaginos* OR candidos* OR candidi* OR thrush OR (vagin* AND candida) OR monili* OR vulvovagin* OR "vaginal infection" OR "vaginal yeast infection" OR "vaginal yeast infections") AND (probiotic OR probiotics) |
| Search results limited with filter "Title Abstract Keyword" |
| Results from both Cochrane Reviews and Trials were used for data selection |

**S2.2 P/I, O criteria and Study Type for Section 2.2** (Phase IIa: identification of RCT studies on efficacious probiotic supplements against BV and VVC)

| **Population** | Inclusion: All female subjects (women) of/after reproductive age (over 13 years of age), including menopausal women, pregnant women, nursing women.  Exclusion: Studies involving female subjects aged under 13 years. |
| --- | --- |
| **Intervention** | Probiotic capsule or powder etc. (i.e. not fermented food) delivered orally (not topical or intravaginal) |
| **Outcome** | BV / VVC treatment, reduction of recurrence  improvement in status / cure from BV/VVC / reduction of recurrence  (eliminate null/negative outcomes) |
| **Types of Study** | - Publications reporting on human intervention (efficacy) studies (e.g., randomised controlled studies, randomised uncontrolled studies, non-randomised controlled studies, other intervention studies) selected - Publications reporting on human observational studies (e.g., cohort studies, case-control studies, cross-sectional studies, other observational studies) are excluded - Reviews (systematic or not) are retained and used to check for potentially missing efficaceous strains - Animal and *in vitro* studies are excluded |

**S2.3 Strains identified as efficacious probiotic organisms and their dispersion in Food Science and Technology category indexed research articles**

| **Taxon** | **Strain Identifier** | **Number of references in tiab screening of Phase IIb^1^** |
| --- | --- | --- |
| *Bifidobacterium bifidum* | UBBB-55 | 0 |
| *Lacticaseibacillus paracasei* (*Lactobacillus paracasei*) | LPC12 | 0 |
|  | MG4272 | 0 |
|  | IMC 502 | 1 |
| *Lacticaseibacillus rhamnosus* (*Lactobacillus rhamnosus*) | TOM 22.8 (DSM33500) | 0 |
|  | GG | 388 |
|  | IMC 501 | 8 |
|  | CBT LR5 | 0 |
|  | GR-1 | 20 |
|  | GMNL-680 | 0 |
|  | GMNL-74 | 0 |
|  | HN001 | 50 |
|  | LRH10 | 0 |
|  | MG4288 | 0 |
|  | UBLR-58 | 0 |
|  | CA15 (DSM 33960) | 0 |
| *Lactiplantibacillus plantarum*  (*Lactobacillus plantarum*) | 57B | 1 |
|  | GMNL-682 | 0 |
|  | IS-10506 | 1 |
|  | LP115 | 1 |
|  | MG989 | 0 |
|  | P17630 | 1 |
|  | UBLP-40 | 0 |
| *Lactobacillus acidophilus* | CBT LA1 | 0 |
|  | GLA-14 | 0 |
|  | GMNL-185 | 0 |
|  | LA-5 | 300 |
|  | NAS | 0 |
|  | UBLA-34 | 0 |
| *Lactobacillus casei* | UBLC-42 | 0 |
|  | IMV B-7280 | 0 |
| *Lactobacillus casei rhamnosus* | LCR35 | 0 |
| *Lactobacillus crispatus* | CTV-05 | 0 |
|  | LG55 | 0 |
|  | LMG S-29995 | 0 |
|  | DSM32715 | 0 |
|  | DSM32716 | 0 |
|  | DSM32717 | 0 |
|  | DSM32718 | 0 |
|  | DSM32719 | 0 |
|  | DSM32720 | 0 |
| *Lactobacillus delbrueckii* subsp. *lactis* | LDL114 | 0 |
| *Lactobacillus gasseri* | 57C | 1 |
|  | TM13 | 0 |
| *Lactobacillus helveticus* | LA25 | 0 |
| *Ligilactobacillus salivarius* (*Lactobacillus salivarius*) | MG242 | 0 |
|  | CECT5713 | 2 |
| *Limosilactobacillus fermentum* (*Lactobacillus fermentum*) | 57A | 1 |
|  | LF26 | 0 |
|  | MG901 | 0 |
|  | UBLF-31 | 0 |
| *Limosilactobacillus reuteri* (*Lactobacillus reuteri*) | CBT LU4 | 1 |
|  | RC-14 | 3 |
|  | UBLRu-87 | 0 |

^1^ Phase IIb search structured to locate strain taxa with variants of the strain identifier in topical search on Web of Science for Food Science and Technology category, limited to research articles from dates 1.1.1970 - 11.04.2025; tiab: title and abstract

**S3.1 Search query for Phase IIb** (Identification of Food Science and Technology studies utilizing identified strains as starter organisms to produce fermented foods)

“Lactobacillus crispatus CTV-05” OR “Lactobacillus crispatus CTV05” OR “Lactobacillus acidophilus LA-5” OR “Lactobacillus acidophilus LA5” OR “Lactobacillus gasseri TM-13” OR “Lactobacillus crispatus LG-55” OR “DSM-32717” OR “DSM-32720” OR “DSM-32718” OR “DSM-32716” OR “Lactobacillus salivarius MG-242” OR “Lactobacillus fermentum MG-901” OR “Lactobacillus plantarum MG-989” OR “Lactobacillus paracasei MG-4272” OR “Lactobacillus rhamnosus MG-4288” OR “Lactobacillus plantarum LP-115” OR “Lactobacillus helveticus LA-25” OR “Lactobacillus rhamnosus LRH-10” OR “Lactobacillus paracasei LPC-12” OR “Lactobacillus fermentum LF-26” OR “Lactobacillus delbrueckii subsp lactis LDL-114” OR “Lactobacillus rhamnosus GG” OR “Lactobacillus crispatus LMG S29995” OR “DSM-32719” OR “DSM-32715” OR “Lactobacillus acidophilus GLA14” OR “Lactobacillus rhamnosus HN-001” OR “Lactobacillus plantarum P-17630” OR “Lactobacillus fermentum 57-A” OR “Lactobacillus plantarum 57-B” OR “Lactobacillus gasseri 57-C” OR “Lactobacillus rhamnosus GR1” OR “Lactobacillus reuteri RC14” OR “Lactobacillus casei rhamnosus LCR-35” OR “Lactobacillus acidophilus UBLA34” OR “Lactobacillus rhamnosus UBLR58” OR “Lactobacillus reuteri UBLRu87” OR “Lactobacillus plantarum UBLP40” OR “Lactobacillus casei UBLC42” OR “Lactobacillus fermentum UBLF31” OR “Bifidobacterium bifidum UBBB55” OR “DSM-33500” OR “Lactobacillus plantarum IS10506” OR “Lactobacillus acidophilus NAS” OR “Lactobacillus gasseri TM13” OR “Lactobacillus crispatus LG55” OR “DSM32717” OR “DSM32720” OR “DSM32718” OR “DSM32716” OR “Lactobacillus salivarius MG242” OR “Lactobacillus fermentum MG901” OR “Lactobacillus plantarum MG989” OR “Lactobacillus paracasei MG4272” OR “Lactobacillus rhamnosus MG4288” OR “Lactobacillus plantarum LP115” OR “Lactobacillus helveticus LA25” OR “Lactobacillus rhamnosus LRH10” OR “Lactobacillus paracasei LPC12” OR “Lactobacillus fermentum LF26” OR “Lactobacillus delbrueckii subsp lactis LDL114” OR “Lactobacillus rhamnosus GG” OR “Lactobacillus crispatus LMG S-29995” OR “DSM32719” OR “DSM32715” OR “Lactobacillus acidophilus GLA-14” OR “Lactobacillus rhamnosus HN001” OR “Lactobacillus plantarum P17630” OR “Lactobacillus fermentum 57A” OR “Lactobacillus plantarum 57B” OR “Lactobacillus gasseri 57C” OR “Lactobacillus rhamnosus GR-1” OR “Lactobacillus reuteri RC-14” OR “Lactobacillus casei rhamnosus LCR35” OR “Lactobacillus acidophilus UBLA-34” OR “Lactobacillus rhamnosus UBLR-58” OR “Lactobacillus reuteri UBLRu-87” OR “Lactobacillus plantarum UBLP-40” OR “Lactobacillus casei UBLC-42” OR “Lactobacillus fermentum UBLF-31” OR “Bifidobacterium bifidum UBBB-55” OR “DSM 33500” OR “Lactobacillus plantarum IS-10506” OR “Lactobacillus acidophilus strain NAS” OR "Lactobacillus casei IMV B-7280" OR "Lactobacillus casei IMVB-7280" OR "Lactobacillus casei IMV B7280" OR "Lacticaseibacillus rhamnosus IMC 501" OR "Lactobacillus rhamnosus IMC 501" OR "Lacticaseibacillus rhamnosus IMC-501" OR "Lacticaseibacillus rhamnosus IMC501" OR "Lacticaseibacillus paracasei IMC 502" OR "Lactobacillus paracasei IMC 502" OR "Lacticaseibacillus paracasei IMC-502" OR "Lacticaseibacillus paracasei IMC502" OR “Lactobacillus acidophilus CBT LA1" OR "Lactobacillus acidophilus CBT-LA1" OR " Lactobacillus acidophilus CBTLA1” OR "Lactobacillus rhamnosus CBT LR5" OR "Lactobacillus rhamnosus CBT-LR5" OR " Lactobacillus rhamnosus CBTLR5” OR “Lacticaseibacillus rhamnosus TOM 22.8” OR "Lactobacillus acidophilus GMNL-185" OR "Lactobacillus acidophilus GMNL185” OR “Ligilactobacillus salivarius CECT5713” OR “Lactobacillus salivarius CECT5713” OR “Ligilactobacillus salivarius CECT-5713” OR “Lactobacillus salivarius CECT-5713” OR "Lactobacillus plantarum GMNL-682" OR " Lactobacillus plantarum GMNL682" OR "Lactobacillus reuteri CBT LU4" OR "Lactobacillus reuteri CBT-LU4" OR " Lactobacillus reuteri CBTLU4” OR “Lactobacillus rhamnosus GMNL-74” OR “Lactobacillus rhamnosus GMNL74” OR “Lactiplanibacillus plantarum LP115” OR “Lactiplanibacillus plantarum LP-115” OR “Lacticaseibacillus rhamnosus LRH10” OR “Lacticaseibacillus rhamnosus LRH-10” OR “Lacticaseibacillus paracasei LPC12” OR “Lacticaseibacillus paracasei LPC-12” OR “Limosilactobacillus fermentum LF26” OR “Limosilactobacillus fermentum LF26” OR “Lactobacillus delbrueckii subsp. Lactis LDL114” OR “Lactobacillus delbrueckii subsp. Lactis LDL114”

**S3.2 Selection criteria for Phase IIb** (Identification of Food Science and Technology studies utilizing identified strains as starter organisms to produce fermented foods)

| **Criteria** | **Key element** |
| --- | --- |
| Article type | Original research article |
| Article content | - Articles utilizing strain(s) in compiled list of probiotic organisms are retained if the strain is used in fermentation of a food matrix. - Any foods prepared for in vitro or in vivo studies are retained. - Any studies where the strain has been added to the matrix however not investigating fermentation (i.e. studies where the strain viability during storage is investigated) are eliminated. |

**S3.3.** **Summary table of food science and technology studies utilizing selected bacteria as fermentation organisms.**

| **Reference** | **Strain^1^** | **Food Matrix** | **Starter Concentration** | **Fermentation and/or Storage Conditions (raw material, final pH, T, etc.)** | **Final Probiotic Count** |
| --- | --- | --- | --- | --- | --- |
| Yao et al., 2014 (62) | LGG (ss) | Dairy | 3% (v/v) | Reconstituted skim milk fermented at 37°C for 4-48 h, storage at 4°C for 3 wk | NA |
| Valík et al., 2008 (63) | LGG (ss) | Dairy | ~3 log CFU/mL | Ultra-pasteurized milk fermented at 6-41°C up to 10 d | 7.6-8.5 log CFU^1^/mL |
| Kim et al., 2025 (64) | LGG (ss) | Dairy | ~9 log CFU/g | Reconstituted skim milk enriched with 2% (w/w) glucose and 1% (w/w) yeast extract fermented at 42°C, 9 h in a water bath, 100 rpm until pH 3.9 | >8 log CFU/g |
| Lauková et al., 2014 (65) | LGG (ss) | Dairy | 6 log CFU/mL | Cow lump cheese ripened for 14 d in cold room | 7-8 log CFU/g |
| Sangwan & Singh, 2018 (66) | LGG (ss) | Dairy | 8 log CFU/mL | Skim milk fermented at 37°C for 12-16 h | 8 log CFU/mL |
| Lim et al., 2021 (67) | LGG (ss) | Dairy | 5% (w/v) | Skim milk fermented at 42°C until pH 3.9 | ~9.2 log CFU/g |
| Lei et al., 2021 (68) | LGG (ss) | Dairy | 1% (w/v) | Skim milk with or without *Dendrobium candidum* extract fermented at 37°C for 48 h | 8.5-8.9 log CFU/mL |
| Suo et al., 2021 (69) | LGG (ss) | Dairy | 6 log CFU/mL | Reconstituted skim milk with different solids contents fermented at 37°C for 6-96 h | 7-9 log CFU/mL |
| Guo et al., 2009 (70) | LGG (ss) | Dairy | 2% (v/v) | Reconstituted skim milk with sugar fermented at 37°C for 24 h, storage at 4°C for 28 d | ~9 log CFU/mL |
| Mitra & Gosh, 2020 (71) | LGG (cw) | Dairy | 2% (w/v) | Milk fermented at 30°C for 20 h, matured at 7–8°C for 10–12 h until pH 4.3, storage for 6 d | 7 log CFU/mL; >7 log CFU/mL after storage |
| Sadera et al., 2024 (72) | LGG (cw) | Dairy | 8 log CFU/mL | Reconstituted whole and skim milk powder and inulin fermented at 43°C until pH 4.5 (~6 h) | NA |
| Settachaimongkon et al., 2014 (73) | LGG (cw) | Dairy | 6 log CFU/g | Reconstituted skim milk fermented to yogurt at 42°C for 4 h, storage at 4°C for 28 d | >6 log CFU/g |
| Cuffia et al., 2017 (74) | LGG (cw) | Dairy | 7.7 log CFU/mL | Pasta filata soft cheese production, 39°C for 3 h, 15 d of ripening | 7.6-8.4 log CFU/g |
| Jia et al., 2016 (75) | LGG (cw) | Dairy | 1-5% (w/v) | Goat milk containing 5-10% sugar fermented at 42°C for 16 h | 8-9 log CFU/mL |
| Settachaimongkon et al., 2015 (76) | LGG (cw) | Dairy | ~6 log CFU/g | Reconstituted skim milk fermented to yogurt at 42°C for 4h, storage at 4°C for 28 d (available in (73)) | 6.4-7.1 log CFU/g after fermentation, 5.4-7.1 log CFU/g during storage |
| Gu et al., 2022 (77) | LGG (cw) | Dairy | 8 log CFU/mL | Milk fermented to yogurt at 42°C until pH 4.5 | >8 log CFU/mL LAB count |
| Rubio et al., 2013 (78) | LGG (ss) | Pork | ~5-7 log CFU/g | Pork sausages fermented at 20-22°C, 90-95% RH^1^ for 6 d, ripened at 12°C, 75-80% RH until a_w_ 0.90-0.93 and pH <5, storage at 1°C for 1 mo | ~8 log CFU/g LAB |
| Gundogan et al., 2023 (79) | LGG (ss) | Poultry | 6 log CFU/mL | Liquid egg white fermented at 37°C for 40-45 h until pH <6 and microwave/oven dried | NA |
| Kocková et al., 2014 (80) | LGG (ss) | Plant-based | 5-6 log CFU/g | Cooked grains (buckwheat, barley, oat, soy, chickpea) fermented at 37°C for 10 h, storage at 5°C for 21 d | 6.7-7.6 log CFU/g |
| Montanari et al., 2020 (81) | LGG (ss) | Plant-based | ~10 log CFU/mL | Peanut and soybean extract supplemented with guava and beet pulp fermented at 37°C for 72 h | >7 log CFU/mL |
| Zhao et al., 2022 (82) | LGG (ss) | Plant-based | 7 log CFU/mL | Bread dough fortified with pre-fermented/unfermented bamboo shoot powder (1:10, w/v) fermented at 37°C for 24 h | NA |
| Kamonsuwan et al., 2024 (83) | LGG (ss) | Plant-based | ~7 log CFU/mL | Black goji berry juice fermented at 37°C for 24 h until pH 3.9 | ~9 log CFU/mL |
| Łopusiewicz et al., 2021 (84) | LGG (ss) | Plant-based | ~6 log CFU/mL | Flaxseed oil cake milk fermented under anaerobic conditions at 42°C for 24 h until pH 3.5-4.4 | 7-8 log CFU/mL |
| Song et al., 2018 (85) | LGG (cw) | Plant-based | 8 log CFU/mL | Adzuki bean milk fermented at 37°C for 36 h under 5% CO_2_ until pH 4.3 | >9 log CFU/mL |
| Hu et al., 2019 (86) | LGG (ss) | Plant-based | 6 log CFU/mL | Carrot juice fermented at 37°C for 48 h | 9 log CFU/mL |
| Chan et al., 2021 (87) | LGG (ss/cw) | Plant-based | ~7 log CFU/mL | Coffee brews fermented at 30°C for 24 h | 6 log CFU/mL |
| Wang et al., 2021 (88) | LGG (ss) | Plant-based | NA | Apple juice fermented at 37°C for 28 h | NA |
| Bhatnagar et al., 2018 (89) | LGG (ss) | Plant-based | ~5 log CFU/mL | Soymilk fermented at 37°C for 24 h | NA |
| Hunaefi et al., 2013 (90) | LGG (cw) | Plant-based | ~6 log CFU/mL | Red cabbage sprouts fermented at 30°C for 12-48 h | >7-8 CFU/mL |
| Chan et al., 2020 (91) | LGG (ss) | Plant-based | ~7 log CFU/mL | Coffee brew with/without glucose and inactivated yeast derivatives fermented at 30°C for 24 h, storage at 4°C for 2-11 wk | ~7 log CFU/mL after fermentation, > 7 log CFU/mL during storage |
| Byresh et al., 2022 (92) | LGG (ss) | Plant-based | 6 log CFU/mL | White finger millet with/without pineapple peel powder solubilized in water fermented at 37°C for 6 h | 8-9 log CFU/mL |
| Zhu et al., 2019 (93) | LGG (ss/cw) | Plant-based | 1-2% (v/v) | Tofu whey fermented at 27-47°C for 36 h | ~9 log CFU/mL |
| Alemneh et al., 2021 (94) | LGG (cw) | Plant-based | 6 log CFU/mL | Slurry made of whole-grain teff flour and water fermented at 30°C for 24 h | 6-8 log CFU/mL |
| Chai et al., 2022 (95) | LGG (cw) | Plant-based | 7.5 log CFU/mL | Blueberry pomace liquid fermented at 37°C for 24 h | NA |
| Pan et al., 2022 (96) | LGG (cw) | Plant-based | ~7 log CFU/mL LAB | Jujube pulp fermented at 37°C for 24 h | >8 log CFU/mL LAB |
| Sigüenza-Andrés et al., 2023 (97) | LGG (cw) | Plant-based | ~7 log CFU/g | Bread flour (ground dried wheat bread) mixed with water and enzymes fermented at 38°C for 24 h | 8-9 log CFU/g |
| Matejceková et al., 2017 (98) | LGG (cw) | Plant-based/Mixed (plant & dairy) | 5.9–6.7 log CFU/mL | Buckwheat mash (buckwheat flour, water/milk, and other ingredients) fermented at 37°C for 8 h, storage at 6 °C for 14 d | 8.4-9.0 log CFU/mL after fermentation, 6.0-8.2 log CFU/mL after storage |
| Li et al., 2016 (99) | LGG (cw) | Mixed (dairy & plant) | ~6 log CFU/g | Reconstituted skim milk without/with Pu-erh tea extract (3, 6 or 9%) fermented at 37°C until pH 4.5 | 9.3-9.6 log CFU/g |
| Picon et al., 2025 (100) | LGG (ss) | Mixed (plant & dairy) | 7.5 log CFU/mL | Coffee cherry extract and milk anaerobically fermented at 37°C for 24 h, storage for 20 d | >8 log CFU/mL |
| Chávez de la Vega et al., 2021 (101) | LGG (ss) | Mixed (plant & dairy) | 6 log CFU/mL | Skim milk supplemented with oat β-glucan fermented at 37°C for 24 h | ~10 log CFU/mL |
| Jaimez-Ordaz et al., 2019 (102) | LGG (ss) | Mixed (plant & dairy) | 6 log CFU/mL | Agave juice or inulin in milk fermented at 37°C for 24 h, storage at 4°C for 21 d | 7.5-8.5 log CFU/mL |
| Seong & Kim, 2021 (103) | LGG (ss) | Mixed (plant & insect) | 7-9 log CFU/mL | Brown rice and brown rice supplemented with cricket (*Gryllus bimaculatus*) semi-solid-state fermentation at 25°C for 70 h | 8-9 log CFU/mL LAB |
| Samtiya et al., 2024 (104) | LGG (ss/cw) | Mixed (dairy & plant) | ̴10 log CFU/mL | Skim milk powder and pearl millet flour solution fermented at 37°C for 8-24 h | NA |
| Escobar et al., 2012 (105) | LGG (ss/cw) | Mixed (plant & dairy) | 8 log CFU/mL | Pavela cheese produced using fresh whole milk with fava bean starch processed at 37-39°C, 1h, pH 6; storage at 4°C for 30 d | ~9 log CFU/g |
| Helland et al., 2004 (106) | LA-5, LGG (ss) | Mixed (plant & dairy) | 7 log CFU/g | Pudding (rice, maize) containing water or milk and other ingredients fermented at 37°C for 12 h until pH 4-5; storage at 4-6°C for 21 d until pH 3-4 | 8–9 log CFU/g |
| Azizishafa et al., 2023 (107) | LGG, LA-5 (ss/cw) | Mixed (cereal & dairy) | 6-7 log CFU/g | Cooked bulgur and milk containing different levels of inulin (0-10%) (doeeneh) fermented at 37°C for 24 h; storage at 4°C for 14 d | 9-10 log CFU/mL after fermentation, 8-9 log CFU/mL after storage |
| Østlie et al., 2003 (108) | LA-5*,* LGG (ss) | Dairy | 1% (v/v) | UHT treated milk fermented at 37°C for 24 h until pH 4 | ~9 log CFU/mL |
| Sadaghdar et al., 2012 (109) | LA-5, HN001 (cw) | Dairy | 8 log CFU/mL | Reconstituted skim milk (6% milk solid-non-fat content) fermented at 42ºC until pH 4.8, storage for 21 d | ~9 log CFU/mL after fermentation, 5-9 log CFU/mL after storage |
| Chaves & Gigante, 2016 (110) | LA-5 (co-culture) | Dairy | ~8 log CFU/g | Cheese (Prato) production, ripening in refrigerated conditions for 1-60 d | 6.6-8.6 log CFU/g |
| Hekmat et al., 2007 (111) | RC-14, GR-1 (cw) | Dairy | 1% (v/v) | Milk fermented at 37°C overnight until pH range 4.3-4.8 | >8 log CFU/mL RC-14; >9 log CFU/mL GR-1 |
| Hekmat et al., 2009 (112) | RC-14, GR-1 (cw) | Dairy | 4% | Low fat milk with 1% fat and 5% sugar fermented at 37°C for 6 h, storage for 28 d | >7 log CFU/mL for GR-1 throughout storage; ~5 log CFU/mL for RC-14 on d 1 |
| Gu et al., 2020 (113) | IMC 502, LGG (cw) | Dairy | 8 log CFU/mL | Milk fermented to yogurt at 42°C until pH 4.5 | >8 log CFU/g LAB count |
| Le & Hekmat, 2020 (114) | GR-1 (cw) | Plant-based | NA | Cooked pulses (black-eyed pea, pigeon pea, kabuli chickpea,desi chickpea) fermented at 37°C for 24-120 h, storage at 4°C for 21 d | >9 log CFU/mL |
| Soltani et al., 2018 (115) | GR-1 (cw) | Mixed (plant & dairy) | 9 log CFU/mL | Fermentation of milk with 3% (w/v) of soaked and autoclaved barley, mung bean or adzuki bean at 38 °C for 6 h, storage at 4°C for 28 d | 8-9 log CFU/mL |
| Aljewicz et al., 2014 (116) | HN001 (cw) | Dairy | ~8.6 log CFU/g | Cheese (Edam) ripened at 12°C and 85% RH for 10 wk | 8-9 log CFU/g |
| Williams & Hekmat, 2017 (117) | GR-1 (ss) | Mixed (plant & dairy) | 8 log CFU/mL | Rice pudding supplemented with inulin and oat fermented at 37°C for 24 h; storage for 21 d | >9 log CFU/mL |
| Huang et al., 2022 (118) | IMC 501, IMC 502 (cc) | Dairy | 7 log CFU/mL | Cheese (Mozzarella) ripened in brine (0.4% NaCl) at 4°C for 30 d until pH 5.8 | 5 log CFU/mL brine |
| Coman et al. 2013 (119) | IMC 501, IMC 502 (ss/cc) | Mixed (plant & dairy) | ~9 log CFU/mL | Buckwheat flour and oat bran in liquid medium (MRS broth without glucose or milk) fermented at 37°C under aerobic conditions for 24 h until pH ~4; storage at 4°C for 4 wk | 7-10 log CFU/mL after fermentation, 9-10 log CFU/mL after storage |
| Dharmasena et al., 2015 (120) | LP115 (ss) | Plant-based | 7 log CFU/g | Oatmeal-coconut water with/without inulin fermented at 27°C for 10 h, storage at 4°C for 7 wk | 7 log CFU/g after fermentation, 6-9 log CFU/g during storage |
| Ibarra et al., 2012 (121) | HN001(cw) | Dairy | ~6 log CFU/mL | Whole milk with skim milk powder, sucrose and other ingredients fermented to yogurt at 37°C until pH 4.6 | >7 log CFU/mL |
| Aljewicz et al., 2014 (122) | HN001 (cw) | Dairy | 8.6 log CFU/g | Cheese (Dutch type) ripened for 6 wk at 12°C and 85% RH | 9.6 log CFU/g |
| Suharja et al., 2014 (123) | HN001 (cw) | Dairy | 6 log CFU/g | Reconstituted whole milk fermented at 30°C until pH 4.12, storage at 12°C/30°C for 5 wk | 6-7 log CFU/g, ~8 log CFU/mL after 1 wk storage |
| Wang et al., 2019 (124) | HN001 (ss) | Plant-based | 7.5 log CFU/mL | Green coffee beans with/without 1% glucose fermented at 37°C for 72 h, pH 6 | 9.5 log CFU/mL (prior roasting) |
| Lu et al., 2018 (125) | HN001 (ss) | Plant-based | ~7 log CFU/mL | Starfruit juice fermented at 30°C for 8 d | 8 log CFU/mL |
| Aljewicz & Cichosz, 2015 (126) | HN001 (cw) | Dairy/Mixed (dairy & plant) | 0.03% (v/v) | Cheese (Dutch type) or cheese-like product produced with milk or milk substituted with palm-fat brined, packaged and ripened 12°C, 85% RH for 6 wk, storage 4°C for 3 mo | 9 log CFU/g |
| Aljewicz & Cichosz, 2015 (127) | HN001 (cw) | Dairy/Mixed (dairy & plant) | 9 log CFU/g | Cheeses (Swiss and Dutch type) produced with milk or milk substituted with palm-based ingredients brined, packaged and ripened for 6 wk at 12°C, 85% RH, storage for 3 mo | 9-10 log CFU/g LAB after ripening, 8-9 log CFU/g after storage |
| Cichosz et al., 2014 (128) | HN001 (cw) | Dairy/Mixed (dairy & plant) | ~8 log CFU/g of cheese | Production of Dutch-type, Swiss-type cheeses and cheese-like products ripened at 12-20°C for 10 wk, storage at 4°C up to 3 mo | 8.2-9.6 log CFU/g during ripening, 7.6-9.9 log CFU/g during storage |
| Oliveira et al, 2001 (129) | LA-5, LCR35 (ss/cw) | Dairy | ~10 log CFU/mL | Milk fermented at 42°C until pH 4.3, storage at 4°C for 7 d | 9 log CFU/mL after fermentation, >7 log CFU/mL during storage |
| Lucas et al., 2004 (130) | LA-5, LCR35 (cw) | Dairy | 0.01% (w/v) | Milk supplemented with skim milk powder and protein hydrolysate fermented at 40°C until pH 4.5, storage at 4°C for 35 d | 3-6 log CFU/mL |
| Dmytrow et al., 2019 (131) | LA-5 (ss) | Dairy | NA | Milk with/without prior transglutaminase treatment and inactivation fermented at 42°C until pH 4.6, storage at 5°C for 14 d | >8 log CFU/mL |
| Meng et al., 1998 (132) | LA-5 (ss) | Dairy | ~6 log CFU/mL | Milk fermented at 37°C until pH 4.5 | >9 log CFU/mL |
| Bimbatti et al., 2024 (133) | LA-5 (ss) | Dairy | ~8 log CFU/mL | Cow milk fermented at 42°C until pH 4.6, storage at 5°C for 28 d | 8-10 log CFU/mL |
| Dabaj et al., 2020 (134) | LA-5 (ss) | Dairy | ~8 log CFU/mL | Whole milk fermented at 37°C for 24 h, ~pH 4.6 | NA |
| Ozturkoglu-Budak et al., 2019 (135) | LA-5 (ss) | Dairy | 8 log CFU/mL | Milk and milk supplemented with inulin fermented at 37°C until pH 4.6 | 8-9 log CFU/g |
| Silva et al., 2021 (136) | LA-5 (ss) | Dairy | 6 log CFU/mL | Whole raw milk fermented at 45°C until pH 4.5, storage at 5°C for 28 d | 6-9 log CFU/mL |
| Diniz-Silva et al, 2020 (137) | LA-5 (ss) | Dairy | 8 log CFU/g | Minas cheese ripened in vacuum packages at 7°C for 21 d | 8-9 log CFU/g |
| Barukčić et al., 2015 (138) | LA-5 (ss) | Dairy | 1% (w/v) | Reconstituted whey fermented at 37°C until pH 4.6 | 9 log CFU/mL |
| Skryplonek et al., 2020 (139) | LA-5 (ss) | Dairy | 6% (w/v) | Whey mixed with milk fermented at 42°C for 7.5 h, storage at 5°C for 21 d | >8.5 log CFU/mL |
| Souza & Saad, 2009 (140) | LA-5 (ss/cw) | Dairy | 6 log CFU/g | Minas cheese ripened at 5°C for 21 d | 6-7 log CFU/g |
| Soares et al., 2019 (141) | LA-5 (ss/cw) | Dairy/Plant-based | 7-8 log CFU/g or mL | Cheese (Requeijão Cremoso) inoculated at 50°C, ripened at 6°C for 45 d, final pH 5.9;  Orange juice inoculated at 50°C, storage at 6°C for 7 d | 6-8 log CFU/g cheese  4-7 log CFU/mL orange juice |
| Didar, 2019 (142) | LA-5 (cw) | Dairy | 2.5% (v/v) | Camel milk fermented at 42°C until pH 4.3, mixed with water (50% w/w) and salt, storage at 4°C for 35 d | 8.2 log CFU/mL after fermentation, 5.1-7.8 CFU/mL during storage |
| Özer & Kirmaci, 2009 (143) | LA-5 (cw) | Dairy | ~9 log CFU/mL | White cheese ripened in brine (12% salt, w/v) for 90 d | 6-8 log CFU/g LAB |
| Azizkhani et al., 2018 (144) | LA-5 (cw) | Dairy | 0.02% (v/v) | Milk and milk supplemented with essential oils fermented to yogurt at 40°C until pH 4.5 (~6h), storage at 4°C for 28 d | ~7 log CFU/mL after fermentation (8-10 log CFU/mL 1 wk storage) |
| Parsa et al., 2015 (145) | LA-5 (cw) | Dairy | 0.5-1.5% (w/v) | Milk and milk enriched with phytosterols fermented to yogurt at 45°C until pH 4.5, storage at 5°C for 2 wk | 6-7 log CFU/g |
| Seyhan et al., 2016 (146) | LA-5 (cw) | Dairy | 0.5% (v/v) | Reconstituted whey containing soy isoflavones /phytosterols (0-1% w/v) fermented at 37°C for 18 h, pH 4.8-5, storage at 4°C for 28 d | 9 log CFU/mL after fermentation, 8 log CFU/mL after storage |
| Silva et al., 2011 (147) | LA-5 (cw) | Dairy | 8 log CFU/g | Cow milk fermented at 30°C for 5h, storage at 12°C 60 d | 8.3-8.5 log CFU/g |
| de Bessa et al., 2015 (148) | LA-5 (cw) | Dairy | >7 log CFU/g | Goat milk supplemented with saccharose and inulin fermented at 43°C until pH 4.6, refrigerated storage for 28 d | >7 log CFU/mL |
| Martín-Diana et al., 2003 (149) | LA-5 (cw) | Dairy | NA | Cow and goat milk fermented at 44°C until a pH 4.5 | 7 log CFU/mL |
| Casarotti & Penna, 2015 (150) | LA-5 (cw) | Dairy | 6 log CFU/mL | Reconstituted skim milk containing apple/banana/grape flour (1%) fermented at 42°C until pH 4.6, storage at 4°C for 28 d | 7 log CFU/mL |
| Vieira et al., 2021 (151) | LA-5 (cw) | Dairy | 14 log CFU/mL | Cow and goat milk fermented at 45°C until pH 4.6, storage at 4°C for 28 d | 6-9 log CFU/mL |
| Niamah, 2019 (152) | LA-5 (cw) | Dairy | 8 log CFU/mL | Skim milk fermented at 37°C for 12 h, storage at 4°C for 12 h | NA |
| Faraki et al., 2020 (153) | LA-5 (cw) | Dairy | 9 log CFU/mL | Milk with/without prebiotic addition fermented to yogurt at 42°C until pH 4.4-4.5, storage at 4°C for 28 d | 7-8 log CFU/g |
| Manzo et al., 2015 (154) | LA-5 (cw) | Dairy | ~7 log CFU/mL | Milk or infant formula with/without prebiotic addition fermented at 40°C until pH 4.7 | 8.6-8.7 log CFU/mL |
| Slacanac et al., 2004 (155) | LA-5 (cw) | Dairy | 5-6 log CFU/mL | Cow and goat milk fermented at 37°C for 8 h | ~7 log CFU/mL |
| Akpinar et al., 2013 (156) | LA-5 (cw) | Dairy | NA | Goat milk with/without prebiotics fermented at 37°C until pH 4.8 for 8 h, storage at 4°C for 21 d | 7-10 log CFU/mL |
| Ribeiro et al.*,* 2014 (157) | LA-5 (cw) | Dairy | 9-10 log CFU/mL | Milk fermented to yogurt at 42°C until pH 4.8, ~3 h, stirred yogurt stored at 5°C 35 d | >8 log CFU/g, 7-8 log CFU/g during storage |
| Nor-Khaizura et al., 2019 (158) | LA-5 (cw) | Dairy | 8 log CFU/mL | Reconstituted skim milk powder fortified with sodium caseinate fermented to yogurt at 22.5-30°C for 7 d | NA |
| Nguyen et al., 2014 (159) | LA-5 (cw) | Dairy | 7 log CFU/g | Raw buffalo milk fermented at 37-43°C until pH of 4.5 | 7 log CFU/g |
| da Cruz Rodrigues et al., 2019 (160) | LA-5 (cw) | Dairy | ~9 log CFU/mL | Reconstituted whole milk powder with sucrose and vanilla extract fermented at 42°C until pH 4.6 | 9 log CFU/g |
| Kocer & Unal, 2018 (161) | LA-5 (cw) | Dairy | ~7 log CFU/mL | Reconstituted skim milk and whole milk fermented at 40°C until pH 4.75 | 6.8-7.5 log CFU/mL |
| Costa et al., 2015 (162) | LA-5 (cw) | Dairy | 9-11 log CFU/g | Goat whole milk with/without prebiotic addition fermented to yogurt at 43°C until pH 4.5, storage for 28 d | 9-11 log CFU/g after fermentation, 8-9 log CFU/g after storage |
| Yilmaztekin et al., 2009 (163) | LA-5 (cw) | Dairy | 9 log CFU/g | Cheese (white cheese) ripened in brine (12% salt) at 4°C up to 90 d | 6-7 log CFU/g |
| Deziderio et al., 2023 (164) | LA-5 (cw) | Dairy/Plant-based | 5.6 log CFU/mL | Oat, rice, almonds, Brazil nut and soybean beverages or milk fermented at 37°C for 12 h, until pH 4-5, storage at 5°C for 28 d | 1-10 log CFU/mL LAB |
| Sertovic et al., 2016 (165) | LA-5 (cw) | Dairy/Plant-based/Mixed | NA | Milk, soy beverage, and their mixtures (75:25, 50:50, 25:75) fermented at 43°C until pH 4.6 | 7.5-8.2 log CFU/mL |
| Shahabbaspour et al., 2012 (166) | LA-5 (cw) | Dairy/Plant-based/Mixed | 8.7 log CFU/mL | Soy milk/milk/mixture fermented at 40°C until pH 4.6, storage at 5°C for 21 d | >8 log CFU/mL LAB |
| Hassani et al., 2015 (167) | LA-5 (ss) | Plant-based | 0.2-0.8 OD_600_ | Sorghum wort fermented at 28-46°C for 18 h | 6-8 log CFU/mL |
| Al-Sahlany & Niamah, 2022 (168) | LA-5 (cw) | Plant-based | 8 log CFU/g | Scallions, white and red onions in brine (5% NaCl) fermented at 37°C for 24 h, pH 4-5, storage for 28 d | 6-8 log CFU/g |
| Bedani et al., 2013 (169) | LA-5 (cw) | Plant-based | 8 log CFU/mL | Soy milk with prebiotic supplement fermented at 37°C until pH 5, storage at 4°C for 28 d | >8 log CFU/mL |
| Hurtado-Murillo et al., 2024 (170) | LA-5 (ss) | Plant-based | 6 log CFU/mL | Quinoa and chickpea flour blended beverages fermented at 38°C and 100 rpm under anaerobic conditions 6–8 h | >8 log CFU/mL |
| Vieira et al., 2021 (171) | LA-5 (cw) | Plant-based | ~8 log CFU/mL | Soy beverages with acerola by-product storage fermented at 37°C for 24 h, storage at 4°C for 28 d | 7 log CFU/mL |
| Hole et al., 2012 (172) | LA-5 (cw) | Plant-based | 7 log CFU/mL | Whole grain barley and oat groat flours mixed with water, fermented at 37°C for 18 h | 9 log CFU/mL |
| Bujna et al., 2018 (173) | LA-5 (cw) | Plant-based | 6 log CFU/mL | Apricot juice (pH adjusted to 6.5) fermented under anaerobic conditions at 37°C for 24 h | 7 log CFU/mL |
| Bedani et al., 2015 (174) | LA-5 (cw) | Plant-based | 9 log CFU/mL | Soy-based products with okara flour fermented at 37°C until pH 4.5 | 8-9 log CFU/mL |
| Eslami-Moshkenani et al., 2016 (175) | LA-5 (cw) | Mixed (plant & dairy) | 0.2 % (w/v) | Heat treated doogh (diluted yogurt, 50% v/v water and NaCl 0.7% w/v) storage at 4°C for 21 d | >7 log CFU/mL during storage |
| Gharibzahedi & Altintas, 2024 (176) | LA-5 (cw) | Mixed (dairy & insect) | 10 log CFU/mL | Skim milk, whey protein and transglutaminase treated mealworm protein isolate fermented to yogurt at 43°C until pH 4.3 | ~7 log CFU/mL |
| Kemsawasd & Chaikham, 2018 (177) | LA-5 (cw) | Mixed (plant & animal) | 11 log CFU/mL | Soy milk supplemented with mulberry leaf extract and other ingredients fermented at 40°C until pH 4.5, storage at 4°C for 30 d | 8-9 log CFU/g after fermentation, 6-9 log CFU/g during storage |
| Vasile et al., 2016 (178) | LA-5 (ss) | Mixed (dairy & plant) | 8 log CFU/mL | Buckwheat flour or oat bran (2- 6%) in liquid medium (MRS broth without glucose, with milk) fermented at 37°C until pH 4.6, storage at 4°C for 28 d | 8.5-9.5 log CFU/mL |
| Ryan et al., 2020 (179) | LA-5 (ss) | Mixed (dairy & plant) | ~6 log CFU/mL | Prefermented milk mixed with mango juice at different ratios (0-40%) fermented at 37°C for 12 h, storage at 4°C for 5 wk | 7.5-8.5 log CFU/mL after fermentation, 3-8 log CFU/mL during storage |
| El-Aidie et al., 2021 (180) | LA-5 (ss) | Mixed (dairy & plant) | 2% (v/v) | Milk containing yellow sweet potato powder fermented at 37°C for 4.5–6 h, storage at 4–6 °C for 14 d | 8-10 log CFU/g |
| Scibisz et al., 2012 (181) | LA-5 (cw) | Mixed (dairy & plant) | 8.0-8.7 log CFU/g | Blueberry preserve and mixed with milk fermented to yogurt at 37°C for 4 h until pH 4.7, storage at 37°C for 8 wk | 8 log CFU/g (6wk), 7 log CFU/g (8wk) |

^1^ **LGG**: *Lacticaseibacillus rhamnosus* (*Lactobacillus rhamnosus*) GG, **GR-1**: *Lacticaseibacillus rhamnosus* (*Lactobacillus rhamnosus*) GR1, **IMC 501**: *Lacticaseibacillus rhamnosus (Lactobacillus rhamnosus)* IMC 501, **HN001**: *Lacticaseibacillus rhamnosus* (*Lactobacillus rhamnosus*) HN001, **LCR35**: *Lactobacillus casei rhamnosus* LCR35, **LA-5**: *Lactobacillus acidophilus* LA-5, **RC-14**: *Limosilactobacillus reuteri* (*Lactobacillus reuteri*) RC-14, **LP115**: *Lactiplantibacillus plantarum (Lactobacillus plantarum)* LP115, **IMC 502**: *Lacticaseibacillus paracasei (Lactobacillus paracasei)* IMC 502, **ss**: single strain, **cc**: co-cultured, **cw**: co-cultured with other bacterial strain(s), **RH**: relative humidity, **CFU**: colony forming units
